# Correlation of powers of Hüsler–Reiss vectors and Brown–Resnick fields, and application to insured wind losses

**DOI:** 10.1007/s10687-023-00474-w

**Published:** 2024-06-14

**Authors:** Erwan Koch

**Affiliations:** 1 Expertise Center for Climate Extremes (ECCE) Faculty of Business and Economics (HEC) - Faculty of Geosciences and Environment, University of Lausanne, Lausanne, Switzerland; 2 Institute of Mathematics, EPFL, Station 8, 1015 Lausanne, Switzerland

**Keywords:** Brown–Resnick random field, Hüsler–Reiss random vector, Insured wind losses, Power damage functions, Reanalysis wind gust data, Spatial dependence, 60G60, 60G70, 62H10, 62H11, 62H20, 62P05, 62P12

## Abstract

Hüsler–Reiss vectors and Brown–Resnick fields are popular models in multivariate and spatial extreme-value theory, respectively, and are widely used in applications. We provide analytical formulas for the correlation between powers of the components of the bivariate Hüsler–Reiss vector, extend these to the case of the Brown–Resnick field, and thoroughly study the properties of the resulting dependence measure. The use of correlation is justified by spatial risk theory, while power transforms are insightful when taking correlation as dependence measure, and are moreover very suited damage functions for weather events such as wind extremes or floods. This makes our theoretical results worthwhile for, e.g., actuarial applications. We finally perform a case study involving insured losses from extreme wind speeds in Germany, and obtain valuable conclusions for the insurance industry.

## Introduction

Extreme-value theory (EVT) offers many statistical techniques and models useful in various fields such as finance, insurance and environmental sciences. Max-stable random vectors (e.g., de Haan and Resnick 1977) naturally arise when extending univariate extreme-value theory to the multidimensional setting, and several parametric multivariate max-stable distributions, such as the Hüsler–Reiss model (Hüsler and Reiss 1989), have been proposed. Max-stable random fields (e.g., de Haan 1984; de Haan and Ferreira 2006; Davison et al. 2012) constitute an infinite-dimensional generalization and are particularly suitable to model the temporal maxima of a given variable at all points in space since they represent the only possible non-degenerate limiting field of pointwise maxima taken over suitably rescaled independent copies of a field (e.g., de Haan 1984). One famous example is the Brown–Resnick field (Brown and Resnick 1977; Kabluchko et al. 2009) which, owing to its flexibility, is generally a good model for spatial extremes of environmental variables. Finite-dimensional distributions of the Brown–Resnick field are Hüsler–Reiss distributions so there is a natural and close link between Hüsler–Reiss vectors and Brown–Resnick fields.

Our main theoretical contributions are explicit formulas for the correlation between powers of the components of bivariate Hüsler–Reiss random vectors, analytical expressions of the spatial correlation function of powers of Brown–Resnick fields, and a careful study of its properties; some results are rather technical to obtain. Studying the correlation function of a field is prominent as it naturally appears when computing the variance of the spatial integral of that field (e.g., Koch 2019b). If the field models an insured cost, its spatial integral models the total insured loss over the integration region, and its variance is thus of interest for any insurance company. The correlation function also explicitly shows up in the standard deviation of the central limit theorem (CLT) of the field, and is thus key for the behaviour of the spatial integral when the size of the integration region becomes large (e.g., Koch 2019b). Moreover, despite its drawbacks, correlation is commonly used in the finance/insurance industry, making its study useful from a practical viewpoint. Finally, the criticism that it does not properly capture extremal dependence is somewhat irrelevant here as we consider the correlation between random variables which already model extreme events.

In a more general setting than EVT, it is often insightful to consider the correlation between various powers of two random variables rather than focusing only on the correlation between these variables. Applying simple non-linear transformations such as the absolute value or powers before taking the correlation sometimes allows one to detect and characterize a strong dependence that would not have been spotted otherwise; this partially alleviates the defect that correlation only captures linear dependence. In finance, it is common to look at the autocorrelation of powers of the absolute values of asset returns. Returns generally do not exhibit any significant autocorrelation (e.g., Cont 2001) whereas their squares or other power values (see, e.g., Ding et al. 1993, who consider powers ranging from 0.125 to 5) show a significantly positive serial correlation.

More importantly for the setting of this work, considering powers (including unity) of random variables is also valuable when these variables are used to model the impact of natural disasters such as, e.g., windstorms or floods. According to physics, the total cost arising from damaging wind to a specific structure should increase as the square (e.g., Simiu and Scanlan 1996) or the cube (e.g., Lamb and Frydendahl 1991; Emanuel 2005; Powell and Reinhold 2007) of the maximum wind speed. Moreover, several studies exploring insured costs have found that power-laws with much higher exponents are appropriate (e.g., Prahl et al. 2012). In the case of flood, the cost is commonly assumed to be proportional to $z/(z+1)$, where $z>0$ is the inundation level measured in meters (e.g., Hinkel et al. 2014; Prahl et al. 2016), which approximately behaves like a power-law with exponent unity for levels much below one meter. Thus, as max-stable vectors and fields are suited to model componentwise and pointwise maxima, studying their powers is worthwhile for assessing costs from extreme wind or flood events. Likewise, powers of random variables naturally arise when modelling the electricity production by wind plants or hydroelectric stations. Wind energy and power available to wind plants are proportional to the cube of wind speed (e.g., Burton et al. 2021) and, similarly, the power of a hydroelectric station is linearly related to the water discharge. Thus, powers of max-stable vectors can be appropriate models for the multivariate extremal production of such generating stations. Note that Brown–Resnick fields have already been used to model extreme wind speeds (e.g., Ribatet 2013; Einmahl et al. 2016) and extreme river discharges (e.g., Asadi et al. 2015). Owing to the ubiquity of power-laws in physics and other domains, powers of max-stable vectors and fields may certainly be suited models for other applications.

In the second part of the paper, we use our theoretical results to study the spatial dependence of insured losses from extreme wind speed for residential buildings over a large part of Germany. We use ERA5 (European Centre for Medium-Range Weather Forecasts Reanalysis 5th Generation) wind speed reanalysis data on 1979–2020 to derive seasonal pointwise maxima, we fit the Brown–Resnick and Smith random fields, and use the appropriate power damage function for the considered region, according to Prahl et al. (2012). The best fitted model leads to a correlation displaying a slow decrease with the distance. We also consider other power values and we find that, for a fixed distance, the correlation between insured costs evolves only slightly with the value of the damage power; this is useful information for insurance companies.

The rest of the paper is organized as follows. Section 2 first briefly reviews Hüsler–Reiss vectors and Brown–Resnick fields, and then details our main theoretical contributions. We present our case study in Section 3, and Section 4 summarizes our main findings and provides some perspectives. All the proofs are gathered in Appendix A. The code and data required to reproduce the results of the case study will be available in a publication on the Zenodo repository. Note that some elements of this article are revised versions of results from Sections 2.2 and 3 and Appendix A of the unpublished work by Koch (2019a). Throughout the paper, ^′^ designates transposition and $\mathbb {N}_*=\mathbb {N} \backslash \{ 0 \}$.

## Theoretical results

### Preliminaries

A random variable *Z* has the standard Fréchet distribution if $\mathbb {P}(Z \le z)=\exp (-1/z), z>0$. A random vector $\varvec{Z}=(Z_1, Z_2)'$ having standard Fréchet marginals is said to follow the bivariate Hüsler–Reiss distribution (Hüsler and Reiss 1989) with parameter $h \in [0, \infty ]$ if

1 $$\begin{aligned} \begin{aligned}&\quad \ \mathbb {P}(Z_1 \le z_1, Z_2 \le z_2) \\&= H(z_1, z_2;\,h) \\&= \exp \left( -\frac{1}{z_2} \Phi \left( \frac{h}{2} - \frac{\log (z_2/z_1)}{h} \right) -\frac{1}{z_1} \Phi \left( \frac{h}{2} - \frac{\log (z_1/z_2)}{h} \right) \right) , \quad z_1, z_2 > 0, \end{aligned} \end{aligned}$$

where $\Phi$ denotes the standard Gaussian distribution function.

This is a popular and flexible distribution for max-stable random vectors, and the parameter *h* interpolates between complete dependence ($h=0$) and independence ($h = \infty$). The *i*-th component, $i=1, 2$, of any bivariate max-stable vector follows the generalized extreme-value (GEV) distribution with location, scale and shape parameters $\eta _i \in \mathbb {R}$, $\tau _i >0$ and $\xi _i \in \mathbb {R}$. If $\varvec{X}=(X_1, X_2)'$ is max-stable with such GEV parameters, then

2 $$\begin{aligned} X_i = \left\{ \begin{array}{ll} \eta _i-\tau _i/\xi _i + \tau _i Z_i^{\xi _i}/\xi _i, &{} \quad \xi _i \ne 0, \\ \eta _i + \tau _i \log Z_i, &{} \quad \xi _i = 0, \end{array} \right. \end{aligned}$$

where $(Z_1, Z_2)'$ is a max-stable vector with standard Fréchet marginal distributions.

In the following, a max-stable random field with standard Fréchet margins will be called simple. The class of Hüsler–Reiss distributions is tightly linked to the Brown–Resnick random field (Brown and Resnick 1977; Kabluchko et al. 2009) which is a flexible and widely used max-stable model. It is very suited to model, e.g., extremes of environmental data (e.g., Davison et al. 2012, Section 7.4, in the case of rainfall) as it allows realistic realizations as well as independence when distance goes to infinity. If $\{ W(\varvec{x}) \}_{\varvec{x} \in \mathbb {R}^d}$ is a centred Gaussian random field with stationary increments and with semivariogram $\gamma _W$, then the Brown–Resnick random field associated with the semivariogram $\gamma _W$ is defined by

3 $$\begin{aligned} Z(\varvec{x}) = \bigvee _{i=1}^{\infty } U_i Y_i(\varvec{x}), \quad \varvec{x} \in \mathbb {R}^d, \end{aligned}$$

where the $(U_i)_{i \ge 1}$ are the points of a Poisson point process on $(0, \infty )$ with intensity function $u^{-2} \textrm{d}u$ and the $Y_i, i\ge 1$, are independent replications of

$$\begin{aligned} Y(\varvec{x})=\exp \left( W(\varvec{x})-\textrm{Var}(W(\varvec{x}))/2 \right) , \quad \varvec{x} \in \mathbb {R}^d, \end{aligned}$$

where $\textrm{Var}$ denotes the variance. It is a stationary^1^ and simple max-stable field whose distribution only depends on the semivariogram (Kabluchko et al. 2009, Theorem 2 and Proposition 11, respectively). Its finite-dimensional distribution functions are Hüsler–Reiss distributions (Kabluchko et al. 2009, Remark 24) and, in particular, for any $\varvec{x}_1, \varvec{x}_2 \in \mathbb {R}^d$,

4 $$\begin{aligned} \mathbb {P}(Z(\varvec{x}_1) \le z_1, Z(\varvec{x}_2) \le z_2) = H \left( z_1, z_2; \sqrt{2 \gamma _W(\varvec{x}_2-\varvec{x}_1)} \right) , \quad z_1, z_2>0. \end{aligned}$$

A commonly used semivariogram is

5 $$\begin{aligned} \gamma _W(\varvec{x})= \left( \Vert \varvec{x} \Vert / \kappa \right) ^{\psi }, \quad \varvec{x} \in \mathbb {R}^d, \end{aligned}$$

where $\kappa >0$ and $\psi \in (0, 2]$ are the range and the smoothness parameters, respectively, and $\Vert \cdot \Vert$ denotes the Euclidean norm. The Smith random field with positive definite covariance matrix $\Sigma$ (Smith 1990) corresponds to the Brown–Resnick field associated with the semivariogram

6 $$\begin{aligned} \gamma _W(\varvec{x})=\varvec{x}' \Sigma ^{-1} \varvec{x}/2, \quad \varvec{x} \in \mathbb {R}^d; \end{aligned}$$

see, e.g., Huser and Davison (2013).

If $\{ X(\varvec{x}) \}_{\varvec{x} \in \mathbb {R}^d}$ is max-stable, there exist functions $\eta (\cdot ) \in \mathbb {R}$, $\tau (\cdot )>0$ and $\xi (\cdot ) \in \mathbb {R}$ defined on $\mathbb {R}^d$, called the location, scale and shape functions, such that

7 $$\begin{aligned} X(\varvec{x}) = \left\{ \begin{array}{ll} \eta (\varvec{x})-\tau (\varvec{x})/\xi (\varvec{x}) + \tau (\varvec{x})Z(\varvec{x})^{\xi (\varvec{x})}/\xi (\varvec{x}), &{} \quad \xi (\varvec{x}) \ne 0, \\ \eta (\varvec{x}) + \tau \log Z(\varvec{x}), &{} \quad \xi (\varvec{x}) = 0, \end{array} \right. \end{aligned}$$

where $\{ Z(\varvec{x}) \}_{\varvec{x} \in \mathbb {R}^d}$ is simple max-stable. In the following, if $\{ X(\varvec{x}) \}_{\varvec{x} \in \mathbb {R}^d}$ is defined by (7) with $\{ Z(\varvec{x}) \}_{\varvec{x} \in \mathbb {R}^d}$ being the Brown–Resnick field associated with the semivariogram $\gamma _W$, then *X* will be referred to as the Brown–Resnick field associated with the semivariogram $\gamma _W$ and with GEV functions $\eta (\varvec{x})$, $\tau (\varvec{x})$ and $\xi (\varvec{x})$. If, for all $\varvec{x} \in \mathbb {R}^d$, $\eta (\varvec{x})=\eta$, $\tau (\varvec{x})=\tau$ and $\xi (\varvec{x})=\xi$, then *X* will be termed the Brown–Resnick field associated with the semivariogram $\gamma _W$ and with GEV parameters $\eta$, $\tau$ and $\xi$.

### Theoretical contributions

Several dependence measures for max-stable vectors and fields have been introduced in the literature: the extremal coefficient (e.g., Schlather and Tawn 2003), the F-madogram (Cooley et al. 2006) and the $\lambda$-madogram (Naveau et al. 2009), among others. Here we propose a new spatial dependence measure which is the correlation of powers of max-stable vectors/fields and not of max-stable vectors/fields themselves. As explained in Section 1, taking power transforms when using correlation is standard practice when dealing with financial time series. For $\varvec{X}$ being defined by (2) with $(Z_1, Z_2)'$ following the Hüsler–Reiss distribution (1), we study $\textrm{Corr}(X_1^{\beta _1}, X_2^{\beta _2})$, where Corr denotes the correlation, and $\beta _i \in \mathbb {N}_*$ such that $\beta _i \xi _i < 1/2$ (to ensure finiteness of the correlation). This allows obtaining the expression of $\textrm{Corr}( X^{\beta (\varvec{x}_1)}(\varvec{x}_1), X^{\beta (\varvec{x}_2)}(\varvec{x}_2))$, $\varvec{x}_1, \varvec{x}_2 \in \mathbb {R}^2$, where *X* is the Brown–Resnick field associated with any semivariogram and with GEV functions $\eta (\varvec{x})$, $\tau (\varvec{x})$, $\xi (\varvec{x})$, and $\beta (\varvec{x})$ is a function taking values in $\mathbb {N}_*$ such that $\beta (\varvec{x}) \xi (\varvec{x}) < 1/2$ for all $\varvec{x} \in \mathbb {R}^2$. If those GEV functions and $\beta (\varvec{x})$ are not spatially constant, the field $\{ X^{\beta (\varvec{x})}(\varvec{x}) \}_{\varvec{x}\in \mathbb {R}^2}$ is not second-order stationary and its correlation function does not only depend on the lag vector. Taking constant GEV and power functions as in the case study is however reasonable when the region considered is fairly homogeneous (in terms, e.g., of elevation, weather influences and distance to a coastline) or not too large. Moreover, every non-stationary random field can be approximated by piecewise stationary fields; see Koch (2019b) and references therein. Therefore, although the most general setting will still be considered for the sake of completeness, our main focus will be on

8 $$\begin{aligned} \mathcal {D}_{X, \beta }(\varvec{x}_1, \varvec{x}_2)=\textrm{Corr} \left( X^{\beta }(\varvec{x}_1), X^{\beta }(\varvec{x}_2) \right) , \quad \varvec{x}_1, \varvec{x}_2 \in \mathbb {R}^2, \end{aligned}$$

where *X* is the Brown–Resnick field with GEV parameters $\eta$, $\tau$, $\xi$, and $\beta \in \mathbb {N}_*$ such that $\beta \xi < 1/2$; in this setting, $X^{\beta }$ is second-order stationary.

On top of being useful for various other applications, powers of rescaled max-stable random fields constitute appropriate models for the field of insured costs from high wind speeds (see Section 3.1 for details) and so (8) can be viewed as the correlation function of insured wind costs, thus being useful for actuarial practice.

Before presenting the main results, we recall the importance of correlation for risk assessment in a spatial context, which justifies studying the correlation despite the existence of dependence measures specifically designed for max-stable fields. Moreover, powers of max-stable fields are not necessarily max-stable themselves, making these measures not directly usable.

Denote by $\mathcal {C}$ the set of all real-valued and measurable^2^ random fields on $\mathbb {R}^2$ having almost surely (a.s.) locally integrable sample paths. Furthermore, let $\mathcal {A}$ denote the set of all compact subsets of $\mathbb {R}^2$ with a strictly positive Lebesgue measure and $\mathcal {A}_c$ be the set of all convex elements of $\mathcal {A}$. For any $A \in \mathcal {A}_c$, let $\varvec{b}_A$ denote its barycenter and $\lambda A$ be the area obtained by applying to *A* a homothety with center $\varvec{b}_A$ and ratio $\lambda >0$.

Let $C \in \mathcal {C}$ model the insured cost per surface unit triggered by events belonging to a specific class (e.g., European windstorms) during a given period of time. The total insured loss on a given region $A \in \mathcal {A}$ can thus be modelled by

$$\begin{aligned} L \left( A, C \right) =\int _A C(\varvec{x}) \textrm{d}\varvec{x}, \end{aligned}$$

and Theorem 4 in Koch (2019b) yields

9 $$\begin{aligned} \textrm{Var}\left( L\left( A, C\right) \right) = \textrm{Var}\left( C(\varvec{0})\right) \int _A \int _A \textrm{Corr} \left( C(\varvec{x}), C(\varvec{y}) \right) \textrm{d}\varvec{x} \textrm{d}\varvec{y}. \end{aligned}$$

Hence the correlation is explicitly involved in the variance of the total insured loss, which is a key quantity for an insurance company.

Moreover, assuming that *C* belongs to $\mathcal {C}$, has a constant expectation and satisfies the CLT (see Koch et al. 2019, Section 3.1) (which holds for $C = X^{\beta }$ if *X* is the Brown–Resnick field associated with the semivariogram (5) and with GEV parameters $\eta$, $\tau$ and $\xi$ such that $\beta \xi < 1/2$),

10 $$\begin{aligned} \sigma = \left[ \textrm{Var}\left( C(\varvec{0})\right) \int _{\mathbb {R}^2} \textrm{Corr} \left( C(\varvec{0}), C(\varvec{x}) \right) \textrm{d}\varvec{x} \right] ^{1/2} \end{aligned}$$

is the standard deviation of the normal distribution appearing in the CLT of *C* and is thus (Koch 2019b, Theorems 2 and 5) essential for the asymptotic distribution of $L(\lambda A, C)$ and the asymptotic properties of spatial risk measures induced by the field *C* and associated with value-at-risk and expected shortfall. The analysis of (8) is thereby insightful for the risk assessment of wind damage; the formulas derived in this paper are used in an ongoing study.

As (2) specifies a transformation of simple max-stable random vectors, we first deal with such vectors. In the next theorem, we take a random vector $\varvec{Z}=(Z_1, Z_2)'$ following the Hüsler–Reiss distribution (1). If $\beta \in \mathbb {R}$ and *Z* is a standard Fréchet random variable, it is easily shown that $Z^{\beta }$ has a finite second moment if and only if $\beta < 1/2$, which imposes, in order for the covariance $\textrm{Cov}(Z_1^{\beta _1}, Z_2^{\beta _2})$ to exist, that $\beta _1, \beta _2 < 1/2$. This covariance and other expressions throughout this section involve, for $\beta _1, \beta _2 < 1/2$,

11 $$\begin{aligned} I_{\beta _1, \beta _2}(h) = \left\{ \begin{array}{ll} \Gamma (1-\beta _1-\beta _2), &{} \text{ if } \quad h=0, \\ \displaystyle \int _{0}^{\infty } \theta ^{\beta _2} \Big [ C_2(\theta ,h) \ C_1(\theta ,h)^{\beta _1+\beta _2 -2} \ \Gamma (2-\beta _1-\beta _2) \\ \qquad + C_3(\theta ,h) \ C_1(\theta ,h)^{\beta _1+\beta _2-1} \ \Gamma (1-\beta _1-\beta _2) \Big ] \textrm{d}\theta , &{} \text{ if } \quad h>0, \end{array} \right. \end{aligned}$$

where $\Gamma$ denotes the gamma function, and, for $\theta , h > 0$,

$$\begin{aligned}&C_1(\theta ,h) = \Phi \left( \frac{h}{2}+ \frac{\log \theta }{h} \right) +\frac{1}{\theta } \Phi \left( \frac{h}{2}- \frac{\log \theta }{h} \right) , C_2(\theta ,h) \\& = \left[ \Phi \left( \frac{h}{2}+ \frac{\log \theta }{h} \right) +\frac{1}{h} \phi \left( \frac{h}{2}+ \frac{\log \theta }{h} \right) -\frac{1}{h \theta } \phi \left( \frac{h}{2}-\frac{\log \theta }{h} \right) \right] \\& \times \left[ \frac{1}{\theta ^2} \Phi \left( \frac{h}{2}- \frac{\log \theta }{h} \right) +\frac{1}{h \theta ^2} \phi \left( \frac{h}{2}- \frac{\log \theta }{h} \right) -\frac{1}{h \theta } \phi \left( \frac{h}{2}+ \frac{\log \theta }{h} \right) \right] , \\& C_3(\theta ,h) = \frac{1}{h^2 \theta } \left( \frac{h}{2}- \frac{\log \theta }{h} \right) \ \phi \left( \frac{h}{2}+ \frac{\log \theta }{h} \right)+\frac{1}{h^2 \theta ^2} \left( \frac{h}{2}+ \frac{ \log \theta }{h} \right) \phi \left( \frac{h}{2}- \frac{\log \theta }{h} \right) , \end{aligned}$$

with $\phi$ denoting the standard Gaussian density function.

We can now state the following result, which is a cornerstone of this section.

#### Theorem 1

Let $\varvec{Z}=(Z_1, Z_2)'$ follow the Hüsler–Reiss distribution (1) with parameter *h*. Then, for all $\beta _1, \beta _2 < 1/2$,

12 $$\begin{aligned} \textrm{Cov} \left( Z_1^{\beta _1}, Z_2^{\beta _2} \right) = I_{\beta _1, \beta _2} \left( h \right) - \Gamma (1-\beta _1) \Gamma (1-\beta _2). \end{aligned}$$

#### Remark 1

Theorem 1 stems from unpublished work in Section 4.5.1 of the PhD thesis by Koch (2014).

We adapt Theorem 1 to the more realistic setting where the margins are general GEV distributions with non-zero shape parameters. The support of such margins possibly includes strictly negative values, and we thus consider powers which are strictly positive integers.

#### Theorem 2

Let $\varvec{Z}$ having (1) as distribution function with parameter *h*, and let $\varvec{X}=(X_1, X_2)'$ be the transformed version of $\varvec{Z}$ by (2) with $\eta _i \in \mathbb {R}$, $\tau _i >0$ and $\xi _i \ne 0$, $i=1, 2$. Moreover, let $\beta _i \in \mathbb {N}_*$ such that $\beta _i \xi _i < 1/2$, $i=1,2$. Then,

13 $$\begin{aligned} \begin{aligned}&\quad \ \textrm{Cov} \left( X_1^{\beta _1}, X_2^{\beta _2} \right) = \sum _{k_1=0}^{\beta _1} \sum _{k_2=0}^{\beta _2} B_{k_1, \beta _1, \eta _1, \tau _1, \xi _1, k_2, \beta _2, \eta _2, \tau _2, \xi _2} \ I_{(\beta _1-k_1)\xi _1, (\beta _2-k_2) \xi _2} \left( h\right) \\&\quad - \sum _{k_1=0}^{\beta _1} \sum _{k_2=0}^{\beta _2} B_{k_1, \beta _1, \eta _1, \tau _1, \xi _1, k_2, \beta _2, \eta _2, \tau _2, \xi _2} \ \Gamma (1-[\beta _1-k_1]\xi _1) \Gamma (1-[\beta _2-k_2]\xi _2), \end{aligned} \end{aligned}$$

where

$$\begin{aligned} \begin{aligned}&\quad \ B_{k_1, \beta _1, \eta _1, \tau _1, \xi _1, k_2, \beta _2, \eta _2, \tau _2, \xi _2} \\ {}&= {\beta _1 \atopwithdelims ()k_1} \left( \eta _1-\frac{\tau _1}{\xi _1} \right) ^{k_1} \left( \frac{\tau _1}{\xi _1} \right) ^{\beta _1-k_1} {\beta _2 \atopwithdelims ()k_2} \left( \eta _2-\frac{\tau _2}{\xi _2} \right) ^{k_2} \left( \frac{\tau _2}{\xi _2} \right) ^{\beta _2-k_2}, \end{aligned} \end{aligned}$$

and, for $$i=1, 2$$,

14 $$\begin{aligned} \begin{aligned} \textrm{Var} \left( X_i^{\beta _i} \right) = \sum _{k_1=0}^{\beta _i} \sum _{k_2=0}^{\beta _i} B_{k_1, k_2, \beta _i, \eta _i, \tau _i, \xi _i}&\{ \Gamma (1-\xi _i[2 \beta _i - k_1 -k_2]) \\&- \Gamma (1-[\beta _i-k_1]\xi _i) \Gamma (1-[\beta _i-k_2]\xi _i) \}, \end{aligned} \end{aligned}$$

where, for $\eta \in \mathbb {R}$, $\tau >0$, $\xi \ne 0$, and $\beta \in \mathbb {N}_*$ such that $\beta \xi < 1/2$,

$$\begin{aligned} B_{k_1, k_2, \beta , \eta , \tau , \xi }= {\beta \atopwithdelims ()k_1} {\beta \atopwithdelims ()k_2} \left( \eta -\frac{\tau }{\xi } \right) ^{k_1+k_2} \left( \frac{\tau }{\xi } \right) ^{2\beta -(k_1+k_2)}. \end{aligned}$$

The combination of (13) and (14) immediately yields the expression of $\textrm{Corr}( X_1^{\beta _1}, X_2^{\beta _2})$. We have assumed in Theorem 2 that $\xi _i \ne 0$ but, as shown now, the case $\xi _1=\xi _2=0$ is easily recovered by taking $\xi _1=\xi _2=\xi$ and letting $\xi$ tend to 0 in (13). Before stating the next result, we recall that the distribution function of any bivariate max-stable vector $(Z_1, Z_2)^{\prime }$ with standard Fréchet margins can be written

$$\begin{aligned} \mathbb {P}(Z_1 \le z_1, Z_2 \le z_2)=\exp (-V(z_1, z_2)), \quad z_1, z_2 >0, \end{aligned}$$

where the function *V*, called the exponent measure, is strictly positive, homogeneous of order $-1$, and satisfies $V(z, \infty )=V(\infty , z)=1/z$ for any $z>0$.

#### Proposition 1

Let $\beta _1, \beta _2 \in \mathbb {N}_*$, $\varepsilon >0$ and $S_{\beta _1, \beta _2, \varepsilon } = \{ \xi \ne 0: \xi <\min \{ 1 /[2 \beta _1 (1+\varepsilon )], 1 /[2 \beta _2 (1+\varepsilon )] \} \}$. Let $\varvec{Z}$ be a simple max-stable vector with continuous exponent measure and let $\varvec{X}_{\xi }=(X_{1, \xi }, X_{2, \xi })'$ be the transformed version of $\varvec{Z}$ by (2) with $\eta _i \in \mathbb {R}$, $\tau _i >0$ and $\xi _i = \xi \in S_{\beta _1, \beta _2, \epsilon }$, $i=1, 2$. Let $\varvec{X}_{0}=(X_{1, 0}, X_{2, 0})'$ be built as $\varvec{X}_{\xi }$ but with $\xi =0$. Then,

$$\begin{aligned} \lim _{\xi \rightarrow 0} \textrm{Cov} \left( X_{1, \xi }^{\beta _1}, X_{2, \xi }^{\beta _2} \right) = \textrm{Cov} \left( X_{1, 0}^{\beta _1}, X_{2, 0}^{\beta _2} \right) . \end{aligned}$$

Using similar arguments, we get $\lim _{\xi \rightarrow 0} \textrm{Var}( X_{i, \xi }^{\beta _i})=\textrm{Var}( X_{i, 0}^{\beta _i})$, which yields

$$\begin{aligned} \lim _{\xi \rightarrow 0} \textrm{Corr} \left( X_{1, \xi }^{\beta _1}, X_{2, \xi }^{\beta _2} \right) = \textrm{Corr} \left( X_{1, 0}^{\beta _1}, X_{2, 0}^{\beta _2} \right) . \end{aligned}$$

This result obviously applies if $\varvec{Z}$ follows the Hüsler–Reiss distribution (1).

Next proposition, which is an immediate corollary of Theorem 2, provides all the necessary ingredients for the computation of our dependence measure $\mathcal {D}_{X, \beta }$ in (8).

#### Corollary 1

Under the same assumptions as in Theorem 2 but with $\eta _1=\eta _2=\eta$, $\tau _1=\tau _2=\tau$, $\xi _1=\xi _2=\xi$ and $\beta _1=\beta _2=\beta$, we have

15 $$\begin{aligned} \begin{aligned}&\quad \ \textrm{Cov} \left( X_1^{\beta }, X_2^{\beta } \right) \\&= g_{\beta , \eta , \tau , \xi } \left( h \right) - \sum _{k_1=0}^{\beta } \sum _{k_2=0}^{\beta } B_{k_1, k_2, \beta , \eta , \tau , \xi } \ \Gamma (1-[\beta -k_1]\xi ) \Gamma (1-[\beta -k_2]\xi ), \end{aligned} \end{aligned}$$

with

16 $$\begin{aligned} g_{\beta , \eta , \tau , \xi }(h) = \sum _{k_1=0}^{\beta } \sum _{k_2=0}^{\beta } B_{k_1, k_2, \beta , \eta , \tau , \xi } \ I_{(\beta -k_1)\xi , (\beta -k_2) \xi } \left( h \right) , \end{aligned}$$

and, for $$i=1, 2$$,

17 $$\begin{aligned} \begin{aligned} \textrm{Var} \left( X_i^{\beta } \right) = \sum _{k_1=0}^{\beta } \sum _{k_2=0}^{\beta } B_{k_1, k_2, \beta , \eta , \tau , \xi }&\{ \Gamma (1-\xi [2 \beta - k_1 -k_2]) \\&- \Gamma (1-[\beta -k_1]\xi ) \Gamma (1-[\beta -k_2]\xi ) \}. \end{aligned} \end{aligned}$$

The following theorem, which is a direct consequence of (4) and Corollary 1, gives the expression of $\mathcal {D}_{X, \beta }$.

#### Theorem 3

Let *X* be the Brown–Resnick field associated with the semivariogram $\gamma _W$ and with GEV parameters $\eta \in \mathbb {R}$, $\tau >0$, $\xi \ne 0$, and let $\beta \in \mathbb {N}_*$ such that $\beta \xi < 1/2$. Then

18 $$\begin{aligned} \mathcal {D}_{X, \beta }(\varvec{x}_1, \varvec{x}_2)=\textrm{Cov} \left( X^{\beta }(\varvec{x}_1), X^{\beta }(\varvec{x}_2) \right) /\textrm{Var}(X^{\beta }(\varvec{0})), \quad \varvec{x}_1, \varvec{x}_2 \in \mathbb {R}^2, \end{aligned}$$

where $\textrm{Cov} \left( X^{\beta }(\varvec{x}_1), X^{\beta }(\varvec{x}_2) \right)$ is given by (15) with $h=\sqrt{2 \gamma _W(\varvec{x}_2-\varvec{x}_1)}$ and $\textrm{Var}(X^{\beta }(\varvec{0}))$ is given by (17).

Note that the case $\xi =0$ is easily recovered as explained above.

#### Remark 2

The combination of (4) and Theorem 2 yields the following more general result than Theorem 3. Let $\{ X(\varvec{x}) \}_{\varvec{x} \in \mathbb {R}^2}$ be the Brown–Resnick field associated with the semivariogram $\gamma _W$ and with GEV functions $\eta (\varvec{x}) \in \mathbb {R}$, $\tau (\varvec{x})>0$, $\xi (\varvec{x}) \ne 0$, and let $\beta (\varvec{x})$ be a function taking values in $\mathbb {N}_*$ such that $\beta (\varvec{x}) \xi (\varvec{x}) < 1/2$ for any $\varvec{x} \in \mathbb {R}^2$. Then,

19 $$\begin{aligned} \begin{aligned}&\quad \ \textrm{Corr} \left( X^{\beta (\varvec{x}_1)}(\varvec{x}_1), X^{\beta (\varvec{x}_2)}(\varvec{x}_2) \right) \\&= \frac{\textrm{Cov} \left( X^{\beta (\varvec{x}_1)}(\varvec{x}_1), X^{\beta (\varvec{x}_2)}(\varvec{x}_2) \right) }{\sqrt{\textrm{Var}(X^{\beta (\varvec{x}_1)}(\varvec{x}_1)) \textrm{Var}(X^{\beta (\varvec{x}_2)}(\varvec{x}_2))}}, \quad \varvec{x}_1, \varvec{x}_2 \in \mathbb {R}^2, \end{aligned} \end{aligned}$$

where $\textrm{Cov} \left( X^{\beta (\varvec{x}_1)}(\varvec{x}_1), X^{\beta (\varvec{x}_2)}(\varvec{x}_2) \right)$ is given by (13) with $h=\sqrt{2 \gamma _W(\varvec{x}_2-\varvec{x}_1)}$, $\eta _i = \eta (\varvec{x}_i), \tau _i = \tau (\varvec{x}_i), \xi _i = \xi (\varvec{x}_i)$, $\beta _i = \beta (\varvec{x}_i)$, $i=1,2$, and $\textrm{Var}(X^{\beta (\varvec{x}_i)}(\varvec{x}_i))$ is given by (17) with $\eta _i = \eta (\varvec{x}_i), \tau _i = \tau (\varvec{x}_i), \xi _i = \xi (\varvec{x}_i)$, $\beta _i = \beta (\varvec{x}_i)$.

The Smith field being a member of the class of Brown–Resnick fields, Theorem 3 and Remark 2 also apply for *X* being the Smith field with any covariance matrix.

The analytical formulas in Theorems 1, 2 and 3, Corollary 1, and Remark 2 allow one to get the true values (up to minor numerical integration errors) of the respective quantities, which is clearly useful in many situations. They also constitute a reference enabling the assessment of approximated computation methods. One is Monte Carlo (MC) estimation, which consists in simulating many realizations of Hüsler–Reiss vectors or Brown–Resnick fields, taking their powers, and computing the empirical covariance or correlation. We assess its performance in a simulation study exposed in Appendix C.1, and show that, in some configurations, the approximation is poor for a number of simulations *S* as large as $10^5$. Increasing *S* to $10^6$ or $10^7$ leads to high computation times compared to those associated with our analytical formulas. Another approximated method is empirical estimation based on the available data. We evaluate its performance in Appendix C.2 and demonstrate that, for a number of temporal observations and sites commonly encountered in applications, the errors are non-negligible and larger than the MC estimator’s ones (for *S* sufficiently large). In a data analysis context, the model parameters must first be estimated before our formulas or MC can be used. Then, the formula-based approach consists in plugging the estimates in our analytical expressions, and the MC approach entails simulating using the obtained parameter estimates. In that context, even our method is not perfectly accurate because of estimation errors on the model parameters, and confidence intervals for the quantity of interest can be derived by combining the delta method and results by Koch and Robert (2022). Nonetheless, owing to the conclusions from Appendix C, we expect the formula-based estimator to outperform the MC estimator and, provided the model is well-specified and *S* is large enough, the MC one to be more accurate than the empirical estimator. We thus recommend the use of the analytical formulas presented above rather than the MC or empirical approaches. If the model is strongly misspecified, the empirical estimator may however obviously outperform both the formula-based and MC ones.

The analytical formulas we propose are valuable also because they enable us to study the mathematical properties of the involved quantities (e.g., their evolution with respect to the various parameters and $\Vert \varvec{x}_2 - \varvec{x}_1 \Vert$) and to obtain analytical expressions of $\textrm{Var}(L(A, C))$ in (9) and $\sigma$ in (10); see a subsequent work about spatial risk measures.

The influence of the marginal parameters and of the power $\beta$ merits some theoretical comments. Let $\varvec{Z}=(Z_1, Z_2)'$ and $\varvec{X}=(X_1, X_2)'$ be as in Theorem 2 and suppose that $X_1$ and $X_2$ are a.s. strictly positive (i.e., $\xi _1, \xi _2>0$ and $\eta _1 - \tau _1/\xi _1, \eta _2 - \tau _2/\xi _2>0$). For $\eta \in \mathbb {R}$, $\tau >0$ and $\xi \ne 0$, the transformation $z \mapsto \eta -\tau /\xi + \tau z^{\xi }/\xi$, $z>0$, is strictly increasing and the same applies for $x \mapsto x^{\beta }$, $x>0$, with $\beta \in \mathbb {N}$, and $z \mapsto z^{\beta ^*}$, $z>0$, with $0< \beta ^* < 1/2$. Thus, owing to the invariance of the copula of a distribution under strictly increasing transformations of the margins, the copula of $(X_1^{\beta _1}, X_2^{\beta _2})'$ is the same whatever the values of $\beta _i \in \mathbb {N}_*$, and is the same as the copula of $(Z_1^{\beta _1^*}, Z_2^{\beta _2^*})'$ whatever the values of $\beta _i^*$ such that $0< \beta _i^* < 1/2$. However, the correlation between two random variables does not only depend on their copula but also on their margins, and is typically not invariant under non-linear transformations. We do not have equality between $\textrm{Corr} ( X_1^{\beta _1}, X_2^{\beta _2})$ and $\textrm{Corr} (Z_1^{\beta _1^*}, Z_2^{\beta _2^*})$ in general, as can also be seen directly from the formulas, and this also holds in the particular case where $\varvec{Z}$, $\varvec{X}$ and $\beta _1, \beta _2$ are as in Corollary 1 and $\beta _1^* = \beta _2^* = \beta ^*$ such that $0<\beta ^*< 1/2$. We have $\textrm{Corr} ( X_1^{\beta }, X_2^{\beta })\ne \textrm{Corr} (Z_1^{\beta ^*}, Z_2^{\beta ^*})$ and, for $\beta \ne 1$, $\textrm{Corr} ( X_1^{\beta }, X_2^{\beta })\ne \textrm{Corr} ( X_1, X_2)$. Thus, $\mathcal {D}_{X, \beta }$ in (8) is not invariant with respect to the marginal parameters $\eta$, $\tau$, $\xi$ and the power $\beta$. Taking the appropriate values of those quantities is necessary when using $\mathcal {D}_{X, \beta }$ for concrete risk assessment problems, and studying its sensitivity with respect to $\beta$ is also of interest. The conclusions of this paragraph regarding the correlations are a fortiori true if $X_1, X_2$ are not a.s. strictly positive; in that case, even the mentioned equalities of copulas do not hold in general.

We now investigate the behaviour of the function $g_{\beta , \eta , \tau , \xi }$ defined in (16) in order to derive useful conclusions about $\mathcal {D}_{X, \beta }$ and because we need it in an ongoing work about spatial risk measures. Next proposition states the strict decreasingness and continuity of $g_{\beta , \eta , \tau , \xi }$, and characterizes its behaviour around 0 and at $\infty$. The proof of Point (i) is appealing as it first involves showing a result (Proposition 3 in Appendix A.6.1) about the correlation order, which is a classical concept of dependence comparison in actuarial risk theory (e.g., Denuit et al. 2005, Section 6.2).

#### Proposition 2

For all $\eta \in \mathbb {R}$, $\tau >0$, $\xi \ne 0$ and $\beta \in \mathbb {N}_*$ such that $\beta \xi < 1/2$, the function $g_{\beta , \eta , \tau , \xi }$ defined in (16)

(i) is strictly decreasing.
(ii) satisfies
  20 $$\begin{aligned} \lim _{h \rightarrow 0} g_{\beta , \eta , \tau , \xi }(h) = \sum _{k_1=0}^{\beta } \sum _{k_2=0}^{\beta } B_{k_1, k_2, \beta , \eta , \tau , \xi } \Gamma (1-\xi [2 \beta - k_1 -k_2]) \end{aligned}$$
  and is continuous everywhere on $[0, \infty )$.
(iii) satisfies
  21 $$\begin{aligned} \lim _{h \rightarrow \infty } g_{\beta , \eta , \tau , \xi }(h)=\sum _{k_1=0}^{\beta } \sum _{k_2=0}^{\beta } B_{k_1, k_2, \beta , \eta , \tau , \xi } \ \Gamma (1-[\beta -k_1]\xi ) \Gamma (1-[\beta -k_2]\xi ). \end{aligned}$$

By Theorem 3, $\mathcal {D}_{X, \beta }(\varvec{x}_1, \varvec{x}_2)$ depends on $\varvec{x}_1$ and $\varvec{x}_2$ through $\gamma _W(\varvec{x}_2 - \varvec{x}_1)$ only. As a variogram is a non-negative conditionally negative definite function, it follows from Berg et al. (1984, Chapter 4, Section 3, Proposition 3.3) that $d(\varvec{x}_1, \varvec{x}_2)= \sqrt{2 \gamma _W(\varvec{x}_2-\varvec{x}_1)}$, $\varvec{x}_1, \varvec{x}_2 \in \mathbb {R}^2$, defines a metric. For many common models of isotropic semivariogram $\gamma _W$, $\gamma _W(\varvec{x}_2-\varvec{x}_1)$ is a strictly increasing function of $\Vert \varvec{x}_2-\varvec{x}_1 \Vert$, which implies by (18) and Proposition 2(i) that $\mathcal {D}_{X, \beta }(\varvec{x}_1, \varvec{x}_2)$ is a strictly decreasing function of $\Vert \varvec{x}_2-\varvec{x}_1 \Vert$; such a decrease of the correlation with the distance seems natural. Moreover (18) and (20) give that $\lim _{ \varvec{x}_2-\varvec{x}_1 \rightarrow \varvec{0}} \mathcal {D}_{X, \beta }(\varvec{x}_1, \varvec{x}_2)=1$, and (18) and (21) imply, provided $\lim _{\Vert \varvec{x}_2-\varvec{x}_1 \Vert \rightarrow \infty } \gamma _W(\varvec{x}_2-\varvec{x}_1)=\infty$, that $\lim _{\Vert \varvec{x}_2-\varvec{x}_1 \Vert \rightarrow \infty } \mathcal {D}_{X, \beta }(\varvec{x}_1, \varvec{x}_2)=0$. The faster the increase of $\gamma _W$ to infinity, the faster the convergence of $\mathcal {D}_{X, \beta }(\varvec{x}_1, \varvec{x}_2)$ to 0. These results are consistent with our expectations. For a function *f* from $\mathbb {R}^2$ to $\mathbb {R}$, by $\lim _{\Vert \varvec{h} \Vert \rightarrow \infty } f(\varvec{h})=\infty$, we mean $\lim _{h \rightarrow \infty } \inf _{\varvec{u} \in \mathcal {B}_1} \{ f(h \varvec{u}) \}=\infty$, where $\mathcal {B}_1= \{ \varvec{x} \in \mathbb {R}^2: \Vert \varvec{x} \Vert =1 \}$.

## Case study

**Fig. 1:**
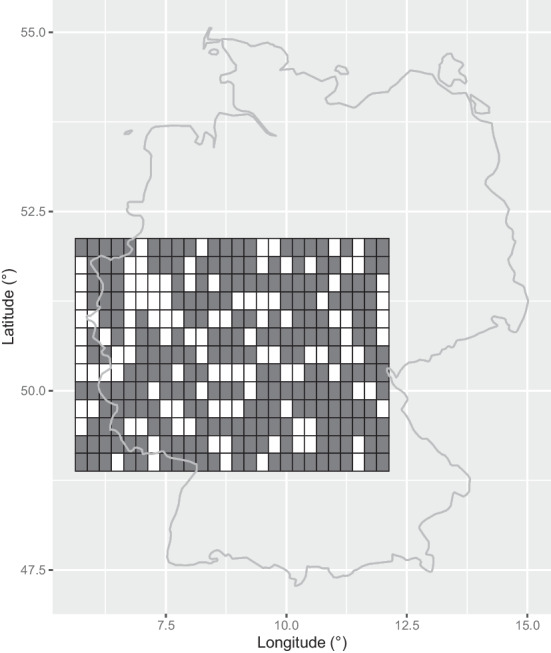
The grey and white cells correspond to the 226 and 118 calibration and validation grid points, respectively

We focus on insured losses from wind extremes for residential buildings over a large part of Germany, more precisely over the rectangle from $5.75 ^\circ$ to $12 ^\circ$ longitude and $49 ^\circ$ to $52 ^\circ$ latitude (see Fig. 1). We apply the results developed in Section 2.2 for assessing the spatial dependence of those losses. For the insured cost field, we use the model introduced in Koch (2017, Section 2.3), that is

22 $$\begin{aligned} C(\varvec{x}) = E(\varvec{x}) D(X(\varvec{x})),\quad \varvec{x} \in \mathbb {R}^2, \end{aligned}$$

where *E* is the strictly positive^3^ and deterministic field of insured value per surface unit, $D: \mathbb {R} \mapsto [0,1]$ is the damage function, and *X* is the model for the random field of the environmental variable generating risk. Applying the damage function *D* to *X* allows getting at each site the insured cost ratio, which, multiplied by the insured value, gives the corresponding insured cost. We assume the risk to be generated by wind speed maxima and we model the latter with a Brown–Resnick and a Smith max-stable model. Section 3.1 outlines and thoroughly justifies the power damage function *D* that we will use. In Section 3.2 we describe the wind speed data and perform model estimation, selection and validation. Finally, we apply in Section 3.3 the results of Section 2.2 using the derived insured cost model.

### Power damage function

We consider the damage function

23 $$\begin{aligned} D(w) = (w/c_1)^{\beta }, \quad w \le c_1, \end{aligned}$$

where $\beta \in \mathbb {N}_*$ and $c_1 >0$. The quantity $c_1$ corresponds to the wind speed for which the insured cost ratio equals unity. We define *D* only for $w \le c_1$ as $c_1$ is typically much larger than the finite upper endpoint of the distribution of wind speed maxima over Germany (see below).

Power functions are well suited to the case of wind. The total cost for a specific structure should increase as the square or the cube of the maximum wind speed since wind loads and dissipation rate of wind kinetic energy are proportional to the second and third powers of wind speed, respectively. For arguments supporting the use of the square, see. e.g., Simiu and Scanlan (1996, Eqs. (4.7.1), (8.1.1) and (8.1.8) and the interpretation following Eq. (4.1.20)). Regarding the cube, see, among others, Lamb and Frydendahl (1991, Chapter 2, p. 7) where the cube of the wind speed appears in the severity index, and Emanuel (2005). In his discussion of the paper by Powell and Reinhold (2007), Kantha (2008) states that wind damage for a given structure must be proportional to the rate of work done (and not the force exerted) by the wind and therefore strongly argues in favour of the cube. In addition to this debate about whether the square or cube is more appropriate for total costs, several studies in the last two decades have found power-laws with much higher exponents when insured costs are considered. For instance, Prahl et al. (2012) find powers ranging from 8 to 12 for insured losses on residential buildings in Germany (local damage functions). Prahl et al. (2015) argue that, if the total cost follows a cubic law but the insurance contract is triggered only when that cost exceeds a strictly positive threshold (e.g., in the presence of a deductible), then the resulting cost for the insurance company is of power-law type but with a higher exponent. We have validated this statement using simulations and observed that the resulting exponent depends on the threshold (not shown).

Several authors (e.g., Klawa and Ulbrich 2003; Pinto et al. 2007; Donat et al. 2011) use, even in the case of insured losses, a cubic relationship that they justify with the physical arguments given above. However, they apply the third power to the difference between the wind speed value and a high percentile of the wind distribution and not to the effective wind speed; as shown by Prahl et al. (2015, Appendix A3), this is equivalent to applying a much higher power to the effective wind speed. Note that exponential damage functions are sometimes also encountered in the literature (e.g., Huang et al. 2001; Prettenthaler et al. 2012); we do not consider such functions here.

According to Prahl et al. (2012) who use (23) as well, a spatially-constant exponent of 10 seems appropriate in our region for insured losses on residential buildings; see their Fig. 2. Finally, (23) yields $c_1 = w/D(w)^{1/\beta }$ for any $w>0$ and one reads in Prahl et al. (2012, Fig. 1) $D(26) \approx 10^{-5}$, leading to $c_1 \approx 82.2$ m s^-1^. Our damage function is then

24 $$\begin{aligned} D(w) = (w/82.2)^{10}, \quad {w \le 82.2}. \end{aligned}$$

As will be seen, the normalization does not play any role in our application.

### Wind data and model for extreme winds

#### Wind data

We consider hourly maxima of the 3 s wind gust at 10 m height (as defined by the World Meteorological Organization) from 1 January 1979 08:00 central European time (CET) to 1 January 2020 at 00:00 CET. This is publicly available data from the European Centre for Medium-Range Weather Forecasts (ECMWF); more precisely we use the “10 m wind gust since previous post-processing” variable in the ERA5 (ECMWF Reanalysis 5th Generation) dataset. The covered region is a rectangle from $5.75 ^\circ$ to $12 ^\circ$ longitude and $49 ^\circ$ to $52 ^\circ$ latitude and the resolution is $0.25 ^\circ$ latitude and $0.25 ^\circ$ longitude, leading to 338 grid points. We randomly choose 226 of them to fit the models and use the remaining 112 for model validation; see Fig. 1. This area encompasses the Ruhr region in Germany and is associated with high residential insured values per surface unit.

We derive at each grid point the 42 seasonal (from October to March) maxima and fit the models to the resulting pointwise maxima. For the first and last season, the maxima are computed over January–March and October–December, respectively. Focusing on October–March allows us to get rid of seasonal non-stationarity in the wind speed time series and to mainly account for winter storms rather than intense summer thunderstorms.

#### Model

We consider both the Brown–Resnick field with semivariogram (5) and the Smith field. As mentioned above, max-stable models are very natural ones for pointwise maxima, and the Brown–Resnick field generally shows good performance on environmental data. We model the location, scale and shape parameters as constant across the region, which is reasonable here (this can be explained by the homogeneity in terms of elevation and weather influences). Using trend surfaces for these parameters rather than fitting them separately at each grid point is standard practice as it reduces parameter uncertainty, allows a joint estimation of all marginal and dependence parameters in a reasonable amount of time and enables prediction at sites where no observations are available. Allowing anisotropy in the semivariogram of the Brown–Resnick model would be pertinent but would not modify our main conclusions. Isotropy already leads to a very satisfying model and makes our dependence measure (8) isotropic in the original space, which facilitates our discussions in Section 3.3.

Both models are fitted using maximum pairwise likelihood (e.g., Padoan et al. 2010) implemented in the fitmaxstab function of the SpatialExtremes R package (Ribatet 2020); marginal and dependence parameters were jointly estimated using the Nelder–Mead algorithm with a relative convergence tolerance of $1.49 \times 10^{-8}$. We then perform model selection by minimization of the composite likelihood information criterion (CLIC); see Varin and Vidoni (2005). According to that criterion, the Brown–Resnick field is the most compatible with the data; see Table 1.

**Table 1:** CLIC values and parameters’ estimates (standard errors inside the brackets) of the Brown–Resnick and Smith models

**Brown–Resnick**	CLIC		$\kappa$	$\psi$	$\eta$	$\tau$	$\xi$
	10’503’932		$3.28\ (1.11)$	$0.83\ (0.06)$	$25.69\ (0.41)$	$3.05\ (0.22)$	$-0.12 \ (0.02)$
**Smith**	CLIC	$\sigma _{11}$	$\sigma _{12}$	$\sigma _{22}$	$\eta$	$\tau$	$\xi$
	10’603’208	$4.17 \ (0.75)$	$-0.17\ (0.05)$	$1.03\ (0.19)$	$25.71\ (0.37)$	$3.07\ (0.20)$	$-0.12\ (0.01)$

**Fig. 2:**
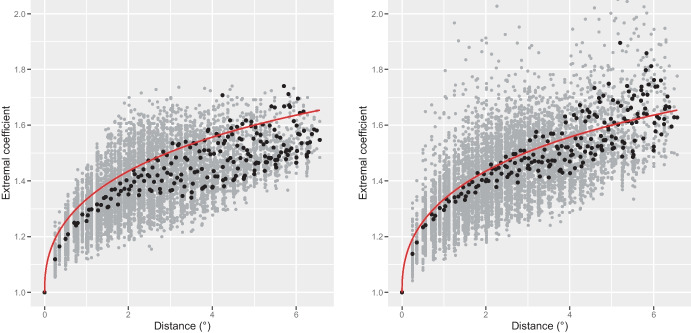
Model’s performance on the validation grid points. Theoretical pairwise extremal coefficient function from the fitted Brown–Resnick model (red line) and empirical pairwise extremal coefficients (dots). The grey and black dots are pairwise and binned estimates, respectively. The empirical extremal coefficients have been computed based on the empirical *F*-madogram using the empirical distribution functions (left) and the obtained GEV parameters (right). The binned estimates have been obtained by first averaging, for any distance, the *F*-madogram estimates over all pairs of grid points at that distance

**Fig. 3:**
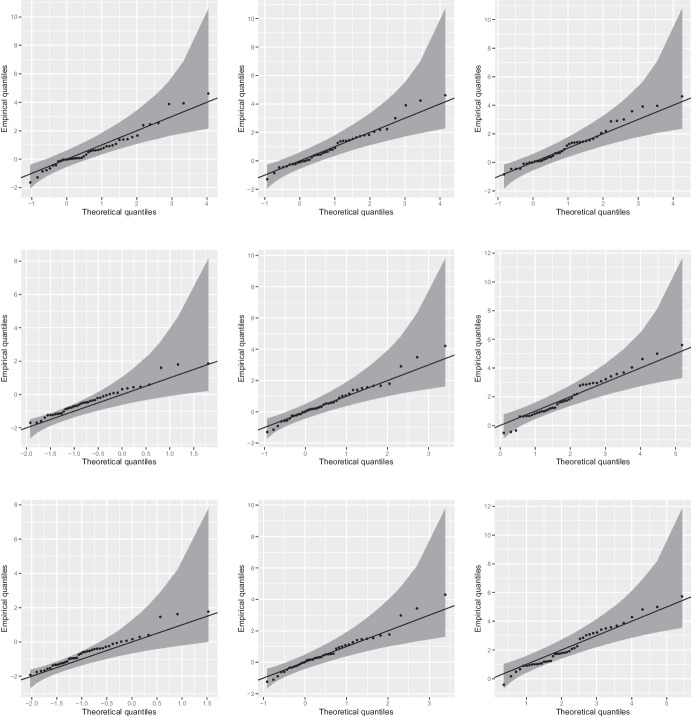
Performance of the fitted Brown–Resnick model on the validation grid points. The top row concerns maxima for pairs of validation grid points separated by a low (left), moderate (middle) and long (right) distance. The middle row focuses on minima (left), mean (middle) and maxima (right) for a group of 40 validation grid points chosen randomly. The bottom row concerns minima (left), mean (middle) and maxima (right) for all 118 validation grid points. Overall envelopes at the 95% confidence level are depicted in dark grey. Note that the data have been transformed to the standard Gumbel scale by using a specific fit of the GEV distribution at each grid point. The theoretical quantiles and envelopes have been obtained by simulating realizations of a simple Brown–Resnick model with parameters $\kappa$ and $\psi$ in Table 1 and taking the logarithm

**Fig. 4:**
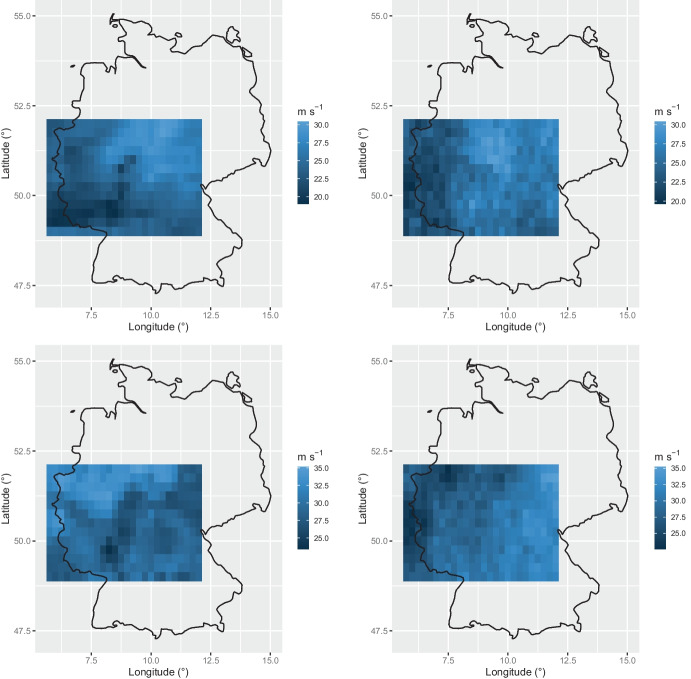
Comparison between observed fields of pointwise maxima and realizations from the fitted Brown–Resnick model. On the left, pointwise maxima over the period October 2005–March 2006 (top) and the period October 2002–March 2003 (bottom). On the right, examples of realizations from the model having values comparable with those in the first column

Although uncomplicated, our choice of constant marginal parameters leads to a decent fit at the marginal level (not shown), and we now mainly discuss the quality of the fit of the dependence structure (joint distribution after normalization to remove the effect of the margins). Figure 2 shows that the theoretical pairwise extremal coefficient function of the fitted Brown–Resnick model agrees reasonably well with the empirical pairwise extremal coefficients for the validation grid points. It is slightly above their binned estimates when those are computed using the empirical distribution functions. This small underestimation of the spatial dependence likely comes from the choice of parsimonious trend surfaces for the location, scale and shape parameters, and disappears when we compute the empirical extremal coefficients using the marginal parameters’ estimates. Overall Fig. 2 indicates that the proposed model fits the pairwise extremal dependence structure of the data fairly well. Figure 3, which is the analog of Fig. 7 in Davison et al. (2012), compares the theoretical (from the fitted Brown–Resnick model) and empirical distributions of groupwise maxima, minima and means for groups of grid points of various sizes (2, 40 and 118 in the first, second, and third rows, respectively). It thus offers a complementary assessment to Fig. 2 as it reflects the quality of the modelled extremal dependence structure in higher dimensions than two. Taking the maximum, minimum and mean as statistics enables us to consider various features of the joint distributions while making the analysis tractable. The fit displayed by Fig. 3 is quite satisfactory. Finally Fig. 4 suggests, for two seasons with different ranges of values, that realizations from our model have similar patterns as observed pointwise maxima, although being slightly rougher. The combination of these goodness-of-fit assessments shows that the proposed model is well suited to our data, and so that this case study is useful in practice.

Having shown that our model performs well, we fit it to the data corresponding to all grid points in order to get as accurate parameters’ estimates as possible; see Table 2. Our estimates are in line with general findings on wind speed extremes. Many studies point out that the shape parameter $\xi$ is usually slightly negative, entailing that the distribution of wind speed maxima has a finite right endpoint. E.g., Ceppi et al. (2008) obtain a $\xi$ ranging from $-0.2$ to 0 by fitting a generalized Pareto distribution (GPD) to in situ observations over Switzerland. Similarly, Della-Marta et al. (2007) fit a GPD to ERA-40 (ECMWF Reanalysis originally intended as a 40-year reanalysis) windstorms data over Europe and find negative values, between $-0.1$ and $-0.3$ on most of land areas; see their Fig. 4.15. Note that the commonly encountered strict negativity of $\xi$ for wind speed maxima makes the condition $\beta \xi <1/2$ in (8) non-restrictive for this kind of application, since $\beta >0$. Typical values for the location and scale parameters $\eta$ and $\tau$ for yearly maxima over Europe are about 25 m s^-1^ and 3 m s^-1^, respectively; e.g., considering annual maxima at 35 weather stations in the Netherlands, Ribatet (2013) obtains trend surfaces whose intercepts are about 27 m s^-1^ for $\eta$ and 3.25 m s^-1^ for $\tau$. Finally, a value of the smoothness parameter $\psi$ between 0.2 and 1 seems reasonable; e.g, Ribatet (2013) obtains 0.24 on the Netherlands data and, on similar ones, Einmahl et al. (2016) find 0.40. We obtain a higher value perhaps because reanalysis data tend to be smoother than in situ observations.

**Table 2:** Parameters’ estimates (standard errors inside brackets) when using all grid points for the fit

$\kappa$	$\psi$	$\eta$	$\tau$	$\xi$
$3.39\ (1.18)$	$0.81\ (0.05)$	$25.71\ (0.41)$	$3.03\ (0.22)$	$-0.12 \ (0.02)$

### Results

Using (22), (23) and the facts that $E(\varvec{x})>0$ for any $\varvec{x} \in \mathbb {R}^2$ and $c_1^{\beta }>0$, we get

$$\begin{aligned} \textrm{Corr}(C(\varvec{x}_1), C(\varvec{x}_2)) = \textrm{Corr} \left( X^{\beta }(\varvec{x}_1), X^{\beta }(\varvec{x}_2) \right) = \mathcal {D}_{X, \beta }(\varvec{x}_1, \varvec{x}_2). \end{aligned}$$

**Fig. 5:**
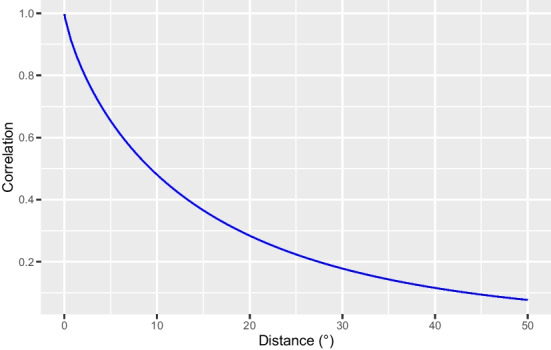
Evolution of $\mathcal {D}_{X, 10}(\varvec{x}_1, \varvec{x}_2)$ with respect to $\Vert \varvec{x}_2-\varvec{x}_1 \Vert$ for *X* being the Brown–Resnick field with semivariogram (5) and parameters in Table 2

Therefore, our dependence measure (8) naturally appears in concrete assessments of the spatial risk associated with extreme wind speed. In this section, we thoroughly study the evolution of $\mathcal {D}_{X, \beta }(\varvec{x}_1, \varvec{x}_2)$ with respect to $\Vert \varvec{x}_2-\varvec{x}_1 \Vert$, where *X* is the Brown–Resnick model fitted to the data in Section 3.2.2, i.e., with semivariogram (5) and parameters in Table 2, and where $\beta$ has the proper value on our region, i.e., 10. The integral in $I_{\beta _1, \beta _2}$ (see (11)) has no closed form and therefore a numerical approximation is required. For this purpose, we use adaptive quadrature with a relative accuracy of $10^{-13}$. Figure 5 shows a decrease of $\mathcal {D}_{X, \beta }$ from 1 to 0 as the Euclidean distance increases, in agreement with our theoretical results of Section 2.2. The decrease is quite slow owing to fairly large range $\kappa$ and rather low smoothness $\psi$. For two sites $5 ^\circ$ and $10 ^\circ$ away, $\mathcal {D}_{X, 10}$ is still as high as 0.65 and 0.48, respectively. The latter conclusion is however hypothetical as the largest distance between two grid points in our region is about $6.93 ^{\circ }$; fitting our model on a wider region would be possible, but the assumption of spatially-constant GEV parameters and power might be less suitable. This slow decrease points out the need for an insurer to cover a wider region than the one considered here in order to benefit from sufficient spatial diversification.

**Fig. 6:**
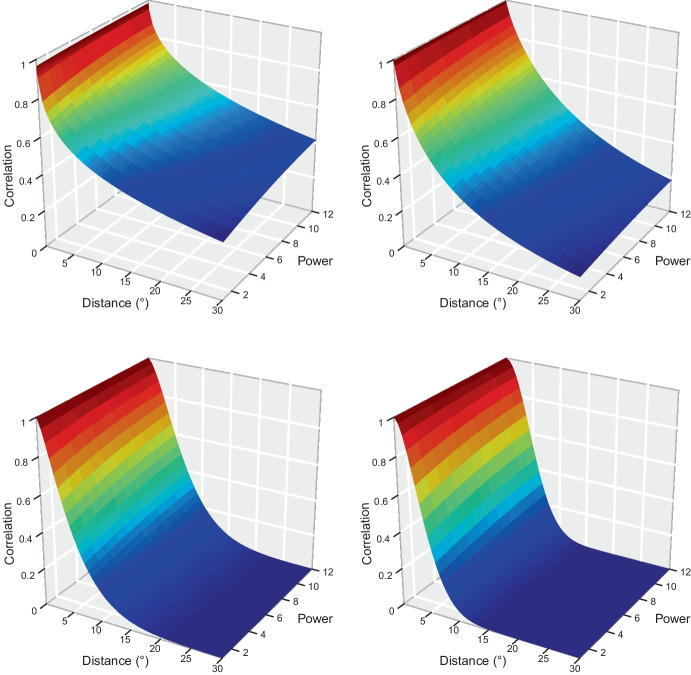
Evolution of $\mathcal {D}_{X, \beta }(\varvec{x}_2-\varvec{x}_1)$ with respect to the distance $\Vert \varvec{x}_2-\varvec{x}_1 \Vert$ and the power $\beta$, where *X* is the Brown–Resnick field with semivariogram (5) with $\psi = 0.5\,\text{(top } \text{ left), } 0.81\,\text{(top } \text{ right), } 1.5\,\text{(bottom } \text{ left) } \text{ and } 2\,\text{(bottom } \text{ right) }$, and whose other parameters are given in Table 2

As already mentioned, various values (basically from 2 to 12) of damage powers have been proposed in the literature and the appropriate one may depend on the insurance contract. Moreover, as explained in Section 1, taking powers (such as the square) of the variables of interest is sometimes worthwhile when using correlation as dependence measure. For example, if the true power is 6, it may also be valuable to study $\textrm{Corr}([X^6(\varvec{x}_1)]^2, [X^6(\varvec{x}_2)]^2)=\textrm{Corr}(X^{12}(\varvec{x}_1), X^{12}(\varvec{x}_2))$. For these reasons, investigating how $\mathcal {D}_{X, \beta }(\varvec{x}_1, \varvec{x}_2)$ varies with $\beta$ for a given max-stable model *X* and various values of $\Vert \varvec{x}_1 - \varvec{x}_2 \Vert$ is useful. Figure 6 shows that whatever the model considered (including the one fitted to our data) and for any given Euclidean distance, $\mathcal {D}_{X, \beta }$ is only faintly sensitive to the value of $\beta$; more precisely, it very slightly increases in a concave way with $\beta$. On top of being potentially insightful for the understanding of max-stable fields, this finding is valuable for actuarial practice as it shows that making a small error on the evaluation of $\beta$ is not very impactful as far as correlation is concerned. Nonetheless this does not imply that the computations should be done with $\beta =1$ regardless of the true power value. First, although evolving little with $\beta$, our dependence measure is not constant with $\beta$ and so using the right value is recommended for accuracy. Second, $\beta$ strongly affects $\textrm{Var}(X^{\beta }(\varvec{x}))$ for any $\varvec{x} \in \mathbb {R}^2$, and thus for instance the covariance function and the variance in (9).

Although the smoothness parameter $\psi$ has been estimated on the data, we also consider various values since $\psi$ heavily affects the rate of decrease of $\mathcal {D}_{X, \beta }$ as the distance between the two sites increases, and thereby the rate of spatial diversification for an insurance company. This allows us to figure out the impact of the use of rougher or smoother data, of estimation error, and of model misspecification. We take $\psi =0.5, 0.81, 1.5, 2$; the value 0.81 is the one we obtained on our data, $\psi =2$ corresponds to the Smith field with $\Sigma =I_2$ (see (6)), $\psi =1.5$ is intermediate between these two settings, and $\psi =0.5$ corresponds to a quite rough field. In accordance with the discussion at the end of Section 2.2, Fig. 6 shows that $\mathcal {D}_{X, \beta }$ decreases from 1 to 0 as the Euclidean distance increases, and this at a higher rate for larger values of $\psi$. The decrease is faster for the Smith field than for all Brown–Resnick fields having $\psi <2$, and if the true value of $\psi$ is close to 0.5 or even 0.81, using the Smith model leads to a serious underestimation of the dependence between insured costs. The minimum Euclidean distance required for $\mathcal {D}_{X, 10}$ to be lower than 0.1 equals $43.60 ^\circ$ for $\psi =0.81$, instead of around $9.54 ^\circ$ for $\psi =2$ (not shown).

The results outlined in the two previous paragraphs remain qualitatively unchanged with other values of $\eta$, $\tau$, $\xi$, and choosing a specific value for $\kappa$ does not induce any loss of generality in our study; should $\kappa$ be different, the appropriate plots would be the same as in Fig. 6 with the values on the x-axis multiplied by the ratio between the true value and the one chosen here.

Finally we briefly study the extension of (8) where the marginal parameters and the power are site-specific. We consider two sites $\varvec{x}_1, \varvec{x}_2$ that are $3 ^{\circ }$ away, but our findings hold more generally. We successively investigate the effects of a spatially-varying power, location, scale and shape; more precisely we evaluate (19) where *X* is the Brown–Resnick model with semivariogram (5)

- with parameters in Table 2 and $\beta (\varvec{x}_1), \beta (\varvec{x}_2) \in \{ 1, \ldots , 12\}$.
- with parameters in Table 2 apart from the location ($\eta (\varvec{x}_1), \eta (\varvec{x}_2) \in [15, 35]$), and $\beta (\varvec{x}_1)=\beta (\varvec{x}_2)=10$.
- with parameters in Table 2 apart from the scale ($\tau (\varvec{x}_1), \tau (\varvec{x}_2) \in [2, 4]$), and $\beta (\varvec{x}_1)=\beta (\varvec{x}_2)=10$.
- with parameters in Table 2 apart from the shape ($\xi (\varvec{x}_1), \xi (\varvec{x}_2) \in [-0.2, -0.06]$), and $\beta (\varvec{x}_1)=\beta (\varvec{x}_2)=10$.

**Fig. 7:**
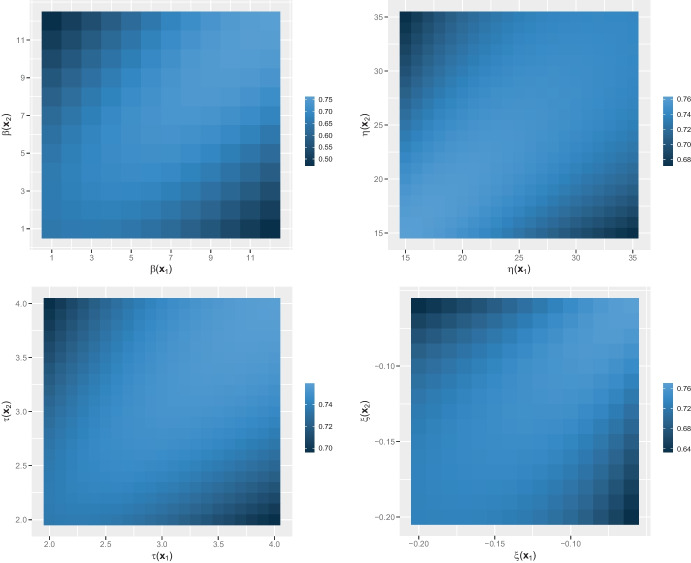
Heatmap of $\textrm{Corr} ( X^{\beta (\varvec{x}_1)}(\varvec{x}_1), X^{\beta (\varvec{x}_2)}(\varvec{x}_2))$, where *X* is the Brown–Resnick field with semivariogram (5) with: parameters in Table 2 and $\beta (\varvec{x}_1), \beta (\varvec{x}_2) \in \{ 1, \ldots , 12\}$ (top left); parameters in Table 2 apart from the location ($\eta (\varvec{x}_1), \eta (\varvec{x}_2) \in [15, 35]$), and $\beta (\varvec{x}_1)=\beta (\varvec{x}_2)=10$ (top right); parameters in Table 2 apart from the scale ($\tau (\varvec{x}_1), \tau (\varvec{x}_2) \in [2, 4]$), and $\beta (\varvec{x}_1)=\beta (\varvec{x}_2)=10$ (bottom left); parameters in Table 2 apart from the shape ($\xi (\varvec{x}_1), \xi (\varvec{x}_2) \in [-0.2, -0.06]$), and $\beta (\varvec{x}_1)=\beta (\varvec{x}_2)=10$

The ranges for the GEV parameters have been chosen to be approximately centred on the estimates obtained on the data. Figure 7 shows that, for a fixed $\beta (\varvec{x}_1)$, the correlation increases with $\beta (\varvec{x}_2)$ on $[1, \beta (\varvec{x}_1)]$ and then decreases. The highest correlation is thus obtained for $\beta (\varvec{x}_2) = \beta (\varvec{x}_1) = \beta$, and, as already seen, slightly increases in a concave way when $\beta$ increases. Also, the higher the difference between $\beta (\varvec{x}_1)$ and $\beta (\varvec{x}_2)$, the lower the correlation. Similar conclusions hold for the scale and shape parameters, although the variations of the correlation are smaller for the chosen range of values. For $\tau (\varvec{x}_1)=\tau (\varvec{x}_2)=\tau$, the increase with respect to $\tau$ is concave, whereas for $\xi (\varvec{x}_1)=\xi (\varvec{x}_2)=\xi$, the increase with respect to $\xi$ is linear. The findings for the location are similar to those for the scale and shape although, for $\eta (\varvec{x}_1)=\eta (\varvec{x}_2)=\eta$, the correlation slowly decreases in a concave way as $\eta$ increases.

#### Remark 3

In practice, site-specific GEV parameters are often modelled through smooth functions of covariates such as latitude, longitude and elevation; see, e.g., Blanchet and Lehning (2010), Davison et al. (2012), and Ribatet (2013). The choice of the appropriate functions and covariates is of course specific to the problem considered.

## Conclusion

Hüsler–Reiss vectors and Brown–Resnick fields are popular and widely used models for componentwise and pointwise maxima. We provide explicit formulas for the correlation between powers of the components of bivariate Hüsler–Reiss vectors and deduce analytical expressions for the correlation function of powers of Brown–Resnick fields. Although extremal models are considered, studying the correlation function makes sense as the latter is required when we are interested in the variance or the asymptotic distribution of the spatial integral of a field, which is typically the case in spatial risk assessment. Moreover, applying power transforms to random variables is relevant for various types of applications, among which the study of impacts due to natural disasters. In the second part of the paper, we use our theoretical contributions and reanalysis wind gust data to study the spatial dependence of modelled insured losses from extreme wind speeds for residential buildings in Germany. We find that the dependence decreases slowly with the distance and that our dependence measure is not very sensitive to the power value.

Although our insured loss model is supported by the literature, thoroughly assessing its performance on insured loss data is prominent for practice, and this is done in an ongoing work. The theoretical results obtained here are used in Koch and Robert (2022) as well as in another current study where spatial risk measures (Koch 2017, 2019b) are applied to concrete assessment of the risk of impacts from extreme wind speeds. Other potentially interesting applications of the derived expressions include flood risk assessment and evaluation of extremal joint electricity production by several wind plants or hydroelectric stations. As estimation is arduous for max-stable fields, it could also be useful to investigate the possibility of estimating the parameters of Hüsler–Reiss distributions and Brown–Resnick random fields by equating the theoretical correlation and the empirical one, and to seek the optimal power value for this purpose. Appendix B, which deals with simple Brown–Resnick fields, can be useful in this respect. Finally, a more detailed study, both theoretically and numerically, of the correlation function expressed in Remark 2 (non-stationary case) would be welcome, and deriving analytical formulas of (8) for other classes of max-stable fields such as the extremal *t* model (Opitz 2013) as well as *r*-Pareto fields (e.g., de Fondeville and Davison 2018) would be useful for applications.

## Figures and Tables

**Fig. 8:**
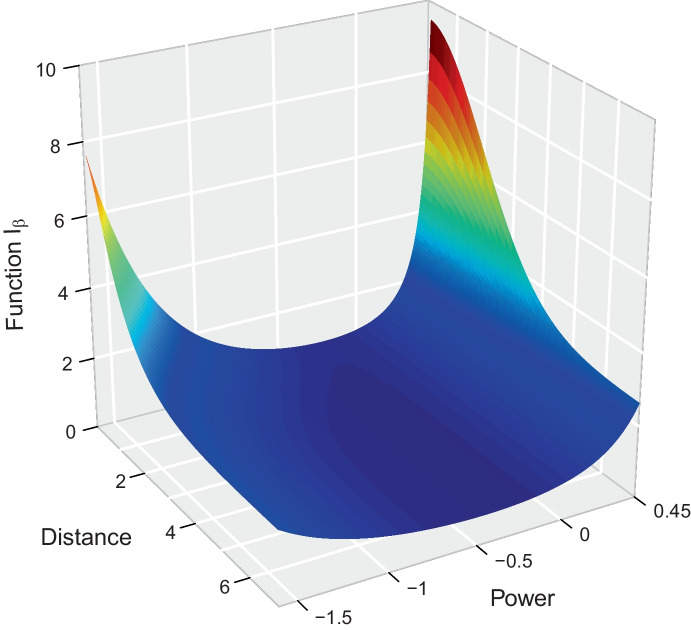
Evolution of the function $I_{\beta }$ with respect to the distance *h* and the power $\beta$ for $\beta \in [-1.6, 0.45]$

## Data Availability

The code and data required to reproduce the results of the case study will be available in a publication on the Zenodo repository.

## Appendix A: Proofs

### A.1 For Theorem 1

#### Proof

First, we show the result for $h=0$. In that case, $Z_1=Z_2$ a.s. (e.g., Hüsler and Reiss 1989, Section 2). Hence, since $Z_1$ and $Z_2$ follow the standard Fréchet distribution, $\mathbb {E} [Z_i^{\beta _i}]= \Gamma (1-\beta _i)$, $i=1, 2$, and thus

$$\begin{aligned} \begin{aligned} \textrm{Cov} \left( Z_1^{\beta _1}, Z_2^{\beta _2} \right)&=\Gamma (1-\beta _1-\beta _2)-\Gamma (1-\beta _1)\Gamma (1-\beta _2) \\&= I_{\beta _1, \beta _2}(0)-\Gamma (1-\beta _1)\Gamma (1-\beta _2). \end{aligned} \end{aligned}$$

Now, we prove the result for $h>0$. We have

$$\begin{aligned} \mathbb {E} \left[ Z_1^{\beta _1} Z_2^{\beta _2} \right] = \displaystyle \int _{0}^{\infty } \displaystyle \int _{0}^{\infty } z_1^{\beta _1} z_2^{\beta _2} l(z_1, z_2) \textrm{d}z_1 \textrm{d}z_2, \end{aligned}$$

where *l* denotes the bivariate density of $\varvec{Z}$. We make the change of variable

$$\begin{aligned} \begin{pmatrix} z_1 \\ z_2 \end{pmatrix} =\begin{pmatrix} u \\ \theta \ u \end{pmatrix} =\begin{pmatrix} \Psi _1(u, \theta ) \\ \Psi _2(u, \theta ) \end{pmatrix} =\Psi (u, \theta ). \end{aligned}$$

The corresponding Jacobian matrix is written

$$\begin{aligned} J_{\Psi }(u, \theta )= \begin{pmatrix} 1 &{} 0 \\ &{} \\ \theta &{} u \end{pmatrix}, \end{aligned}$$

and its determinant is thus $\det (J_{\Psi }(u, \theta ))=u$. Therefore, introducing

$$\begin{aligned} a(z_1,z_2)=z_1^{\beta _1} z_2^{\beta _2} l(z_1, z_2), \quad z_1, z_2 >0, \end{aligned}$$

we have

A1 $$\begin{aligned} \begin{aligned} \mathbb {E} \left[ Z_1^{\beta _1} Z_2^{\beta _2} \right]&= \int _{0}^{\infty } \int _{0}^{\infty } a(z_1,z_2) \textrm{d}z_1 \textrm{d}z_2 \\ {}&= \int \int _{\Psi ^{-1}((0, \infty )^2)} a(\Psi (u, \theta )) \det (J_{\Psi }(u, \theta )) \textrm{d}u\textrm{d}\theta \\ {}&= \int _{0}^{\infty } \int _{0}^{\infty } u^{\beta _1} \theta ^{\beta _2} u^{\beta _2} l(u, \theta u) u \textrm{d}u \textrm{d}\theta \\ {}&= \int _{0}^{\infty } \int _{0}^{\infty } u^{\beta _1+\beta _2 +1 } \theta ^{\beta _2} l(u, \theta u) \textrm{d}u \textrm{d}\theta . \end{aligned} \end{aligned}$$

Differentiation of (1) yields (see, e.g., Padoan et al. 2010, Eq. (4))), for $z_1, z_2>0$,

A2 $$\begin{aligned} \begin{aligned} l(z_1, z_2) =\exp \left( -\frac{\Phi (w)}{z_1}-\frac{\Phi (v)}{z_2} \right) \times \bigg [&\left( \frac{\Phi (w)}{z_1^2}+\frac{\phi (w)}{h z_1^2}-\frac{\phi (v)}{h z_1 z_2} \right) \\&\times \left( \frac{\Phi (v)}{z_2^2}+\frac{\phi (v)}{h z_2^2}-\frac{\phi (w)}{h z_1 z_2} \right) \\ {}&+\left( \frac{v \phi (w)}{h^2 z_1^2 z_2}+\frac{w \phi (v)}{h^2 z_1 z_2^2} \right) \bigg ], \end{aligned} \end{aligned}$$

where

$$\begin{aligned} w=\frac{h}{2}+\frac{\log \left( z_2 / z_1 \right) }{h} \quad \text{ and } \quad v=\frac{h}{2}-\frac{\log \left( z_2/z_1 \right) }{h}. \end{aligned}$$

Therefore, for any $u, \theta >0$,

A3 $$\begin{aligned} \begin{aligned} l(u, \theta u)&= \exp \left( - \frac{1}{u} \left[ \Phi \left( \frac{h}{2}+ \frac{\log \theta }{h} \right) +\frac{1}{\theta } \Phi \left( \frac{h}{2} - \frac{\log \theta }{h} \right) \right] \right) \times \bigg \{ \frac{1}{u^4} \bigg [ \Phi \left( \frac{h}{2}+\frac{\log \theta }{h} \right) \\ {}&\ \ \ +\frac{1}{h} \phi \left( \frac{h}{2}+ \frac{\log \theta }{h} \right) -\frac{1}{h \theta }\phi \left( \frac{h}{2}-\frac{\log \theta }{h} \right) \bigg ] \times \bigg [ \frac{1}{\theta ^2} \Phi \left( \frac{h}{2}- \frac{\log \theta }{h} \right) \\ {}&\ \ \ +\frac{1}{h \theta ^2} \phi \left( \frac{h}{2}- \frac{\log \theta }{h} \right) -\frac{1}{h \theta } \phi \left( \frac{h}{2}+ \frac{\log \theta }{h} \right) \bigg ] \\&\ \ \ + \frac{1}{u^3} \bigg [ \frac{1}{h^2 \theta } \left( \frac{h}{2}- \frac{\log \theta }{h} \right) \ \phi \left( \frac{h}{2}+ \frac{\log \theta }{h} \right) \\ {}&\ \ \ + \frac{1}{h^2 \theta ^2} \left( \frac{h}{2}+ \frac{\log \theta }{h} \right) \ \phi \left( \frac{h}{2}-\frac{\log \theta }{h} \right) \bigg ] \bigg \} \\ {}&= \exp \left( - \frac{C_1(\theta ,h)}{u} \right) \left( \frac{C_2(\theta ,h)}{u^4} + \frac{C_3(\theta ,h)}{u^3} \right) . \end{aligned} \end{aligned}$$

We denote by $\mathcal {F}_{s_f}$ the Fréchet distribution with shape and scale parameters 1 and $s_f>0$, i.e., if $X \sim \mathcal {F}_{s_f}$, $\mathbb {P}(X \le x)= \exp ( - s_f/x ), x > 0.$ Using (A1) and (A3) and the fact that the density of $X \sim \mathcal {F}_{s_f}$ is $l_f(x)=s_f/x^2 \exp \left( -s_f/x \right)$, we obtain

A4 $$\begin{aligned} \begin{aligned}&\quad \ \mathbb {E} \left[ Z_1^{\beta _1} Z_2^{\beta _2} \right] \\&= \int _{0}^{\infty } \theta ^{\beta _2} \left( \int _{0}^{\infty } u^{\beta _1+\beta _2 +1} \exp \left( - \frac{C_1(\theta ,h)}{u} \right) \left( \frac{C_2(\theta ,h)}{u^4} + \frac{C_3(\theta ,h)}{u^3} \right) \textrm{d}u \right) \textrm{d}\theta \\ {}&= \int _{0}^{\infty } C_2(\theta ,h) \ \theta ^{\beta _2} \left( \int _{0}^{\infty } u^{\beta _1+\beta _2 -3 } \exp \left( - \frac{C_1(\theta ,h)}{u} \right) \textrm{d}u \right) \textrm{d}\theta \\ {}&\ \ \ + \int _{0}^{\infty } C_3(\theta ,h) \ \theta ^{\beta _2} \left( \int _{0}^{\infty } u^{\beta _1+\beta _2 -2 } \exp \left( - \frac{C_1(\theta ,h)}{u} \right) \textrm{d}u \right) \textrm{d}\theta \\ {}&= \int _{0}^{\infty } C_2(\theta ,h) \ \theta ^{\beta _2} \left( \int _{0}^{\infty } u^{\beta _1+\beta _2 -1 }\ \frac{1}{u^2}\ \exp \left( - \frac{C_1(\theta ,h)}{u} \right) \textrm{d}u \right) \textrm{d}\theta \\ {}&\ \ \ + \int _{0}^{\infty } C_3(\theta ,h) \ \theta ^{\beta _2} \left( \int _{0}^{\infty } u^{\beta _1+\beta _2} \ \frac{1}{u^2} \exp \left( - \frac{C_1(\theta ,h)}{u} \right) \textrm{d}u \right) \textrm{d}\theta \\ {}&= \int _{0}^{\infty } \frac{C_2(\theta ,h)}{C_1(\theta ,h)} \ \theta ^{\beta _2} \ \mu _{ \beta _1 + \beta _2 -1} \left( \mathcal {F}_{C_1(\theta ,h)} \right) \textrm{d}\theta \\&\quad \ + \int _{0}^{\infty } \frac{C_3(\theta ,h)}{C_1(\theta ,h)} \ \theta ^{\beta _2} \ \mu _{ \beta _1 + \beta _2} \left( \mathcal {F}_{C_1(\theta ,h)} \right) \textrm{d}\theta , \end{aligned} \end{aligned}$$

where $\mu _k(F)$ stands for the *k*-th moment of a random variable having *F* as distribution. It is immediate to see that $\mu _k ( \mathcal {F}_{s_f} ) =s_f^k \ \Gamma (1-k)$, which, combined with (A4), yields the result.□

### A.2 For Theorem 2

#### Proof

Using (2) and the binomial theorem, we obtain

$$\begin{aligned} \begin{aligned}&\quad \ \textrm{Cov} \left( X_1^{\beta _1}, X_2^{\beta _2} \right) \\ {}&= \sum _{k_1=0}^{\beta _1} \sum _{k_2=0}^{\beta _2} {\beta _1 \atopwithdelims ()k_1} \left( \eta _1-\frac{\tau _1}{\xi _1} \right) ^{k_1} \left( \frac{\tau _1}{\xi _1} \right) ^{\beta _1-k_1} {\beta _2 \atopwithdelims ()k_2} \left( \eta _2-\frac{\tau _2}{\xi _2} \right) ^{k_2} \left( \frac{\tau _2}{\xi _2} \right) ^{\beta _2-k_2} \\&\qquad \qquad \times \textrm{Cov} \left( Z_1^{(\beta _1-k_1) \xi _1}, Z_2^{(\beta _2-k_2) \xi _2} \right) , \end{aligned} \end{aligned}$$

which directly yields (13) by Theorem 1.

If *Z* is standard Fréchet, $\mathbb {E}(Z^{\beta ^*})=\Gamma (1-\beta ^*)$ for any $\beta ^* < 1/2$, which gives, for $\beta _1^*, \beta _2^* < 1/2$,

$$\begin{aligned} \textrm{Cov}(Z^{\beta _1^*}, Z^{\beta _2^*})= \Gamma (1 - [\beta _1^* + \beta _2^*]) - \Gamma (1 - \beta _1^*) \Gamma (1 - \beta _2^*). \end{aligned}$$

Using this together with (2) and the binomial theorem yields (14).□

### A.3 For Proposition 1

#### Proof

For $i=1,2$, $X_{i, \xi }$ follows the GEV distribution with parameters $\eta _i$, $\tau _i$ and $\xi$, the density of which we denote by $f_i$. Let us assume that $\xi \in S_{\beta _1, \beta _2, \varepsilon }$ and $\xi >0$. We have for all $\alpha >0$

A5 $$\begin{aligned} \mathbb {E}[\vert X_{i, \xi } \vert ^{\alpha }] = \int _{\eta _i-\tau _i/\xi }^{0} \vert x \vert ^{\alpha } f_i(x) \textrm{d}x + \int _{0}^{\infty } x^{\alpha } f_i(x) \textrm{d}x \end{aligned}$$

and thus

A6 $$\begin{aligned} \sup _{\xi \in \mathcal {S}} \mathbb {E}[\vert X_{i, \xi } \vert ^{\alpha }] \le \sup _{\xi \in \mathcal {S}} \int _{\eta _i-\tau _i/\xi }^{0} \vert x \vert ^{\alpha } f_i(x) \textrm{d}x + \sup _{\xi \in \mathcal {S}} \int _{0}^{\infty } x^{\alpha } f_i(x) \textrm{d}x \end{aligned}$$

for any subset $\mathcal {S}$ of $(0, \infty )$. We deal with the second integral in (A5), for which there is a potential problem at $\infty$. We have

A7 $$\begin{aligned} \begin{aligned}&\quad \int _{0}^{\infty } x^{\alpha } \exp \left( -[1+\xi (x-\eta _i)/\tau _i]^{-1/\xi } \right) [1+\xi (x-\eta _i)/\tau _i]^{-1/\xi -1} \textrm{d}x \\&=\int _{0}^{1} \left[ \eta _i + \tau _i(z^{-\xi }-1)/\xi \right] ^{\alpha } \exp (-z) \textrm{d}z, \end{aligned} \end{aligned}$$

where we used the change of variable $z=[1+\xi (x-\eta _i)/\tau _i]^{-1/\xi }$. As $[ \eta _i + \tau _i(z^{-\xi }-1)/\xi ] \underset{z \rightarrow 0}{\sim }\ \tau _i z^{-\xi }/\xi$, (A7) is finite provided $\alpha \xi <1$. Choose $0<\xi ^*<1/\alpha$, such that (A7) computed at $\xi ^*$ is finite. Introducing $g(\xi )= (z^{-\xi }-1)/\xi$, $\xi >0$, where $z\ge 0$, we have

$$\begin{aligned} \frac{\textrm{d}g(\xi )}{\textrm{d}\xi }=\frac{z^{-\xi }(\log (z^{-\xi }) - 1)}{\xi ^2} + \frac{1}{\xi ^2}. \end{aligned}$$

A well-known inequality states that $\log (z^{-\xi })\ge 1 - 1 / z^{-\xi }$ for any $z\ge 0$, which yields $z^{-\xi }(\log (z^{-\xi }) - 1) \ge -1$ and thus $g'(\xi )\ge 0$. Combined with the fact that $0 \le z \le 1$, this gives for any $0 < \xi \le \xi ^*$

$$\begin{aligned} \begin{aligned} \left| \left[ \eta _i + \tau _i(z^{-\xi }-1)/\xi \right] ^{\alpha } \exp (-z) \right|&= \left[ \eta _i + \tau _i(z^{-\xi }-1)/\xi \right] ^{\alpha } \exp (-z) \\&\le \left[ \eta _i + \tau _i(z^{-\xi ^*}-1)/\xi ^* \right] ^{\alpha } \exp (-z) \end{aligned} \end{aligned}$$

and therefore, taking $\alpha =\beta _i (1 + \varepsilon )$,

$$\begin{aligned} \begin{aligned}&\quad \ \sup _{\xi \in (0, \xi ^*]} \int _{0}^{1} \left[ \eta _i + \tau _i(z^{-\xi }-1)/\xi \right] ^{\beta _i (1 + \varepsilon )} \exp (-z) \textrm{d}z \\ {}&= \int _{0}^{1} \left[ \eta _i + \tau _i(z^{-\xi ^*}-1)/\xi ^* \right] ^{\beta _i (1 + \varepsilon )} \exp (-z) \textrm{d}z < \infty . \end{aligned} \end{aligned}$$

Combining this result with a similar reasoning for the first integral in (A5) and using (A6) yields $\sup _{\xi \in (0, K]}\mathbb {E}[ \vert X_{i, \xi }^{\beta _i} \vert ^{1+\varepsilon }]< \infty$ for some $K>0$. Now, let $Y_{\xi }=X_{1, \xi }^{\beta _1} X_{2, \xi }^{\beta _2}$ and $Y_{0}=X_{1, 0}^{\beta _1} X_{2, 0}^{\beta _2}$. By Cauchy–Schwarz inequality,

$$\begin{aligned} \sup _{\xi \in (0, K]} \mathbb {E}\left[ \left| Y_{\xi } \right| ^{1+\varepsilon } \right] \le \sqrt{\sup _{\xi \in (0, K]} \mathbb {E} \left[ \left| X_{1, \xi }^{\beta _1} \right| ^{2(1+\varepsilon )}\right] } \sqrt{\sup _{\xi \in (0, K]} \mathbb {E} \left[ \left| X_{2, \xi }^{\beta _2} \right| ^{2(1+\varepsilon )}\right] } < \infty . \end{aligned}$$

It follows from Billingsley (1999, p. 31) that the $(X_{1, \xi })_{\xi }$, $(X_{2, \xi })_{\xi }$ and $(Y_{\xi })_{\xi }$ are uniformly integrable for $\xi$ around 0 (from the right).

Now, it is well-known that $X_{i, \xi } \overset{d}{\rightarrow }\ X_{i, 0}$, $i=1, 2$, which implies by the continuous mapping theorem that $X_{i, \xi }^{\beta _i} \overset{d}{\rightarrow }\ X_{i, 0}^{\beta _i}$. Moreover, for any $z_1, z_2 \in \mathbb {R}$,

$$\begin{aligned} \begin{aligned} \mathbb {P} \left( \left[ Z_1^{\xi }-1\right] /\xi \le z_1, \left[ Z_2^{\xi }-1 \right] /\xi \le z_2 \right)&= \mathbb {P} \left( Z_1 \le (1+\xi z_1)^{1/\xi }, Z_2 \le (1+\xi z_2)^{1/\xi } \right) \\&= \exp \left( -V \left( [1+\xi z_1]^{1/\xi }, [1+\xi z_2]^{1/\xi } \right) \right) , \end{aligned} \end{aligned}$$

and

$$\begin{aligned} \mathbb {P} \left( \log Z_1 \le z_1, \log Z_2 \le z_2 \right) = \exp (-V(\exp (z_1), \exp (z_2))), \end{aligned}$$

where *V* is the exponent measure of $(Z_1, Z_2)'$. Thus, by continuity of *V*,

$$\begin{aligned} \lim _{\xi \rightarrow 0} \mathbb {P} \left( \left[ Z_1^{\xi }-1\right] /\xi \le z_1, \left[ Z_2^{\xi }-1\right] /\xi \le z_2 \right) = \mathbb {P} \left( \log Z_1 \le z_1, \log Z_2 \le z_2 \right) , \end{aligned}$$

and therefore

$$\left( \left[ Z_1^{\xi }-1\right] /\xi , \left[ Z_2^{\xi }-1\right] /\xi \right) ' \overset{d}{\rightarrow }\ \left( \log Z_1, \log Z_2 \right) '.$$

Consequently, the continuous mapping theorem yields

$$\begin{aligned} (X_{1, \xi }^{\beta _1}, X_{2, \xi }^{\beta _2})' \overset{d}{\rightarrow }\ (X_{1, 0}^{\beta _1}, X_{2, 0}^{\beta _2})', \end{aligned}$$

and hence, applied again, $Y_{\xi } \overset{d}{\rightarrow }\ Y_{0}$. Finally, Theorem 3.5 in Billingsley (1999) yields that $\lim _{\xi \rightarrow 0} \mathbb {E}(X^{\beta _i}_{i, \xi }) = \mathbb {E}(X^{\beta _i}_{i, 0})$, $i=1,2$ and $\lim _{\xi \rightarrow 0} \mathbb {E}(Y_{\xi })=\mathbb {E}(Y_{0})$. The result follows immediately. Similar arguments give the same conclusion for $\xi <0$.□

### A.4 For Corollary 1

#### Proof

The result is an immediate consequence of Theorem 2.□

### A.5 For Theorem 3

#### Proof

Under the stated assumptions, $X^{\beta }$ is second-order stationary and thus, for any $\varvec{x}_1, \varvec{x}_2 \in \mathbb {R}^2$,

$$\begin{aligned} \textrm{Var}(X^{\beta }(\varvec{x}_1))= \textrm{Var}(X^{\beta }(\varvec{x}_2)) = \textrm{Var}(X^{\beta }(\varvec{0})), \end{aligned}$$

which yields

$$\begin{aligned} \mathcal {D}_{X, \beta }(\varvec{x}_1, \varvec{x}_2)=\textrm{Corr} \left( X^{\beta }(\varvec{x}_1), X^{\beta }(\varvec{x}_2) \right) =\textrm{Cov} \left( X^{\beta }(\varvec{x}_1), X^{\beta }(\varvec{x}_2) \right) /\textrm{Var}(X^{\beta }(\varvec{0})). \end{aligned}$$

By (4), we know that $(X(\varvec{x}_1), X(\varvec{x}_2))^{\prime }$ can be written as the transformed version of $\varvec{Z}$ by (2) with $\eta _1=\eta _2=\eta \in \mathbb {R}$, $\tau _1 = \tau _2 = \tau >0$ and $\xi _1 = \xi _2 = \xi \ne 0$, where $\varvec{Z}$ has (1) as distribution function with parameter $h=\sqrt{2 \gamma _W(\varvec{x}_2-\varvec{x}_1)}$. It follows from Corollary 1 that $\textrm{Cov}( X^{\beta }(\varvec{x}_1), X^{\beta }(\varvec{x}_2))$ is given by (15). Moreover, since $X(\varvec{0})$ follows the GEV distribution with parameters $\eta \in \mathbb {R}$, $\tau >0$ and $\xi \ne 0$, $X^{\beta }(\varvec{0})$ follows the same distribution as $X^{\beta }_1$ and $X^{\beta }_2$ in Corollary 1 and hence has the same variance given by (17).□

### A.6 For Proposition 2(i)

In this section, we denote by $F_{X}$ the distribution function of any random variable *X* and by $F_{X_1, X_2}$ the distribution function of any random vector $\varvec{X}=(X_1, X_2)'$.

#### A.6.1 Preliminary result

We first need the following result.

##### Proposition 3

Let $\varvec{X}=(X_1, X_2)'$ and $\varvec{Y}=(Y_1, Y_2)'$ be random vectors such that $F_{X_1}=F_{Y_1}$ and $F_{X_2}=F_{Y_2}$. We have

$$\begin{aligned} \begin{aligned}&\quad \ \ \ F_{X_1, X_2}(z_1, z_2)< F_{Y_1, Y_2}(z_1, z_2) \ \text{ for } \text{ all } z_1, z_2 > 0 \\&\Longrightarrow \textrm{Cov}(f_1(X_1), f_2(X_2)) < \textrm{Cov}(f_1(Y_1), f_2(Y_2)), \end{aligned} \end{aligned}$$

for all strictly increasing functions $f_1: (0, \infty ) \rightarrow \mathbb {R}$ and $f_2: (0, \infty ) \rightarrow \mathbb {R}$, provided the covariances exist.

##### Proof

The proof is partly inspired from the proof of Theorem 1 in Dhaene and Goovaerts (1996). Let $f_1: (0, \infty ) \rightarrow \mathbb {R}$ and $f_2: (0, \infty ) \rightarrow \mathbb {R}$ be strictly increasing functions. Assume that, for all $z_1, z_2>0$,

A8 $$\begin{aligned} F_{X_1, X_2}(z_1, z_2) < F_{Y_1, Y_2}(z_1, z_2). \end{aligned}$$

We have

$$\begin{aligned} \mathbb {P}(f_1(X_1) \le z_1, f_2(X_2) \le z_2)= \mathbb {P} \left( X_1 \le f_1^{-1}(z_1), X_2 \le f_2^{-1}(z_2) \right) \end{aligned}$$

and the same equality for $\varvec{Y}$. Consequently, since, for all $z_1, z_2>0$, $f_1^{-1}(z_1), f_2^{-1}(z_2)>0$, it follows from (A8) that, for all $z_1, z_2>0$,

A9 $$\begin{aligned} \mathbb {P}(f_1(X_1) \le z_1, f_2(X_2) \le z_2) < \mathbb {P}(f_1(Y_1) \le z_1, f_2(Y_2) \le z_2). \end{aligned}$$

Since $X_1$ and $Y_1$ have the same distribution and this also holds for $X_2$ and $Y_2$, we deduce that

A10 $$\begin{aligned} f_1(X_1) \overset{d}{=}\ f_1(Y_1) \quad \text{ and } \quad f_2(X_2) \overset{d}{=}\ f_2(Y_2). \end{aligned}$$

Using (A9), (A10) and Lemma 1 in Dhaene and Goovaerts (1996), we obtain

$$\begin{aligned} \begin{aligned} \textrm{Cov}(f_1(X_1), f_2(X_2))&=\int _{0}^{\infty } \int _{0}^{\infty } \left[ F_{f_1(X_1), f_2(X_2)}(u,v)-F_{f_1(X_1)}(u) F_{f_2(X_2)}(v)\right] \textrm{d}u \textrm{d}v \\&< \int _{0}^{\infty } \int _{0}^{\infty } \left[ F_{f_1(Y_1), f_2(Y_2)}(u,v)-F_{f_1(Y_1)}(u) F_{f_2(Y_2)}(v) \right] \textrm{d}u \textrm{d}v \\&= \textrm{Cov}(f_1(Y_1), f_2(Y_2)). \end{aligned} \end{aligned}$$

□

#### A.6.2 Proof of Proposition 2(i)

##### Proof

Let $\varvec{Z}=(Z_1, Z_2)'$ be a random vector having the Hüsler–Reiss distribution function (1) with parameter *h*. We immediately obtain that, for all $z_1, z_2>0$,

A11 $$\begin{aligned} \begin{aligned}&\quad \ \frac{\partial \mathbb {P}(Z_1 \le z_1, Z_2 \le z_2)}{\partial h}(h) \\&= \exp \left( -\frac{1}{z_1} \Phi \left( \frac{h}{2} + \frac{1}{h} \log \left( \frac{z_2}{z_1} \right) \right) -\frac{1}{z_2} \Phi \left( \frac{h}{2} + \frac{1}{h} \log \left( \frac{z_1}{z_2} \right) \right) \right) T_2, \end{aligned} \end{aligned}$$

where

$$\begin{aligned} \begin{aligned} T_2 =&-\frac{1}{z_1} \left( \frac{1}{2} - \frac{\log (z_2/z_1)}{h^2} \right) \phi \left( \frac{h}{2}+\frac{\log (z_2/z_1)}{h} \right) \\&- \frac{1}{z_2} \left( \frac{1}{2} + \frac{\log (z_2/z_1)}{h^2} \right) \phi \left( \frac{h}{2}-\frac{\log (z_2/z_1)}{h} \right) . \end{aligned} \end{aligned}$$

For all $z_1, z_2>0$, we introduce $y=z_2/z_1$, which is strictly positive. We have

$$\begin{aligned} \begin{aligned} T_2&= \frac{1}{z_2} \bigg [-\frac{z_2}{z_1} \left( \frac{1}{2} - \frac{\log (z_2/z_1)}{h^2} \right) \phi \left( \frac{h}{2}+\frac{\log (z_2/z_1)}{h} \right) \\ {}&\qquad \quad \ - \left( \frac{1}{2} + \frac{\log (z_2/z_1)}{h^2} \right) \phi \left( \frac{h}{2}-\frac{\log (z_2/z_1)}{h} \right) \bigg ] \\ {}&= \frac{1}{z_2} \left[ -y \left( \frac{1}{2} - \frac{\log y}{h^2} \right) \phi \left( \frac{h}{2}+\frac{\log y}{h} \right) - \left( \frac{1}{2} + \frac{\log y}{h^2} \right) \phi \left( \frac{h}{2}-\frac{\log y}{h} \right) \right] \\ {}&= \frac{1}{\sqrt{2 \pi } z_2} \exp \left( -\frac{h^2}{8}-\frac{(\log y)^2}{2h^2} \right) \left[ -y \left( \frac{1}{2} - \frac{\log y}{h^2} \right) y^{-1/2} - \left( \frac{1}{2} + \frac{\log y}{h^2} \right) y^{1/2} \right] \\ {}&= -\frac{y^{1/2}}{\sqrt{2 \pi } z_2} \exp \left( -\frac{h^2}{8}-\frac{(\log y)^2}{2h^2} \right) , \end{aligned} \end{aligned}$$

which is strictly negative. Thus, (A11) gives that, for all $h \ge 0$ and $z_1, z_2>0$,

A12 $$\begin{aligned} \partial \mathbb {P}(Z_1 \le z_1, Z_2 \le z_2)/\partial h(h) <0. \end{aligned}$$

Let us consider $h_1>h_2>0$, and $\varvec{Z}_{1}=(Z_{1, 1}, Z_{1, 2})'$ and $\varvec{Z}_{2}=(Z_{2, 1}, Z_{2, 2})'$ following the Hüsler–Reiss distribution (1) with parameters $h_1$ and $h_2$, respectively. We get from (A12) that $F_{Z_{1, 1}, Z_{1, 2}}(z_1, z_2) < F_{Z_{2, 1}, Z_{2, 2}}(z_1, z_2)$ for all $z_1, z_2 >0$. Since the components of $\varvec{Z}_{1}$ and $\varvec{Z}_{2}$ all follow the standard Fréchet distribution, we have $F_{Z_{1, 1}}=F_{Z_{2, 1}}$ and $F_{Z_{1, 2}}=F_{Z_{2, 2}}$. Now, as $\tau >0$, for $\xi \ne 0$, the function

$$\begin{aligned} \begin{array}{cccc} f: &{} (0, \infty ) &{} \rightarrow &{} \mathbb {R} \\ &{} z &{} \mapsto &{} \left( \eta -\tau /\xi + \tau z^{\xi }/\xi \right) ^{\beta } \end{array} \end{aligned}$$

is strictly increasing. Hence, letting

$$\begin{aligned} Y_{i, j} = \eta -\frac{\tau }{\xi } + \frac{\tau }{\xi } {Z_{i, j}}^{\xi }, \quad i, j=1, 2, \end{aligned}$$

Proposition 3 yields

A13 $$\begin{aligned} \textrm{Cov} \left( Y_{1, 1}^{\beta }, Y_{1, 2}^{\beta } \right) < \textrm{Cov} \left( Y_{2, 1}^{\beta }, Y_{2, 2}^{\beta } \right) . \end{aligned}$$

Furthermore, we know from (15) that, for $i=1, 2$,

A14 $$\begin{aligned} \textrm{Cov} \left( Y_{i, 1}^{\beta }, Y_{i, 2}^{\beta } \right) =g_{\beta , \eta , \tau , \xi }(h_i)-\sum _{k_1=0}^{\beta } \sum _{k_2=0}^{\beta } B_{k_1, k_2, \beta , \eta , \tau , \xi } \ \Gamma (1-[\beta -k_1]\xi ) \Gamma (1-[\beta -k_2]\xi ). \end{aligned}$$

Finally the combination of (A13) and (A14) gives that $g_{\beta , \eta , \tau , \xi }(h_1)<g_{\beta , \eta , \tau , \xi }(h_2)$, showing the result.□

### A.7 For Proposition 2(ii)

#### Proof

Let *X* be the Brown–Resnick field associated with the semivariogram $\gamma _W(\varvec{x})=\Vert \varvec{x}\Vert ^2/2$, $\varvec{x} \in \mathbb {R}^2$, and with GEV parameters $\eta$, $\tau$, and $\xi \ne 0$, and $\beta \in \mathbb {N}_*$ such that $\beta \xi < 1/2$. It is well-known that *X* is sample-continuous.

The field $X^{\beta }$ is stationary by stationarity of *X* and has a finite second moment since $\beta \xi < 1/2$. Accordingly, $X^{\beta }$ is second-order stationary. Moreover, $X^{\beta }$ is sample-continuous and thus, by the same arguments as in the proof of Proposition 1 in Koch et al. (2019), continuous in quadratic mean. Hence, the covariance function of $X^{\beta }$ is continuous at the origin. It implies by Theorem 3 that

$$\begin{aligned} \begin{aligned}&\quad \lim _{\varvec{x} \rightarrow \varvec{0}} \textrm{Cov} \left( X^{\beta }(\varvec{0}), X^{\beta }(\varvec{x}) \right) \\&=\lim _{\varvec{x} \rightarrow \varvec{0}} \left( g_{\beta , \eta , \tau , \xi } \left( \Vert \varvec{x} \Vert \right) - \sum _{k_1=0}^{\beta } \sum _{k_2=0}^{\beta } B_{k_1, k_2, \beta , \eta , \tau , \xi } \ \Gamma (1-[\beta -k_1]\xi ) \Gamma (1-[\beta -k_2]\xi ) \right)&= \textrm{Var} \left( X^{\beta }(\varvec{0}) \right) , \end{aligned} \end{aligned}$$

which, combined with (17), yields (20). This easily gives $\lim _{h \rightarrow 0} g_{\beta , \eta , \tau , \xi }(h)= g_{\beta , \eta , \tau , \xi }(0)$, which implies that $g_{\beta , \eta , \tau , \xi }$ is continuous at $h=0$. The continuity of $g_{\beta , \eta , \tau , \xi }$ at any $h>0$ comes from the fact that the covariance function of a field which is second-order stationary can be discontinuous only at the origin.□

### A.8 For Proposition 2(iii)

#### A.8.1 Preliminary results

##### Lemma 1

Let $\{ X(\varvec{x}) \}_{\varvec{x} \in \mathbb {R}^2}$ be a measurable max-stable random field with GEV parameters $\eta \in \mathbb {R}$, $\tau >0$ and $\xi \ne 0$. Let $\beta \in \mathbb {N}_*$ such that $\beta \xi < 1$. Then, the random field $X^{\beta }$ belongs to $\mathcal {C}$.

##### Proof

The field $X^{\beta }$ is obviously measurable. Furthermore, as *X* has identical univariate marginal distributions, the function $\varvec{x} \mapsto \mathbb {E} [ \vert X(\varvec{x})^{\beta } \vert ]$ is constant and hence locally integrable. Therefore, Proposition 1 in Koch (2019b) yields that $X^{\beta }$ has a.s. locally integrable sample paths.□

Let $\mathcal {B}(\mathbb {R})$ and $\mathcal {B}((0, \infty ))$ denote the Borel $\sigma$-fields on $\mathbb {R}$ and $(0, \infty )$, respectively.

##### Lemma 2

Let $\{ Z(\varvec{x}) \}_{\varvec{x} \in \mathbb {R}^2}$ be a simple max-stable random field. Let $\eta \in \mathbb {R}$, $\tau >0$, $\xi \in \mathbb {R}$ and $\beta \in \mathbb {N}_*$. The function defined by

A15 $$\begin{aligned} D_{\beta , \eta , \tau , \xi }(z)= \left\{ \begin{array}{ll} \left( \eta -\tau /\xi + \tau z^{\xi }/\xi \right) ^{\beta }, &{} \quad \xi \ne 0, \\ \left( \eta + \tau \log z \right) ^{\beta }, &{} \quad \xi = 0, \end{array} \qquad z >0, \right. \end{aligned}$$

is measurable from $((0, \infty ), \mathcal {B}((0,\infty )))$ to $(\mathbb {R},\mathcal {B}(\mathbb {R}))$ and strictly increasing. Moreover, if $\beta \xi <1/2$, then $\mathbb {E} [ \vert D_{\beta , \eta , \tau , \xi }(Z(\varvec{0})) \vert ^{2+\delta } ]<~\infty$ for any $\delta$ such that $0< \delta < 1/(\xi \beta )-2$.

##### Proof

The fact that *D* is measurable and strictly increasing is obvious. Denoting $Y= [D_{\beta , \eta , \tau , \xi }(Z(\varvec{0}))]^{1/\beta }$, we have, for $\delta >0$,

$$\begin{aligned} \mathbb {E} \left[ \left| D_{\beta , \eta , \tau , \xi }(Z(\varvec{0})) \right| ^{2+\delta } \right] =\mathbb {E} \left[ \left| Y^{\beta } \right| ^{2+\delta }\right] = \mathbb {E}\left[ \left| Y \right| ^{\beta (2+\delta )}\right] , \end{aligned}$$

which is finite (see the proof of Proposition 1) provided $\beta (2+\delta ) \xi < 1$ as *Y* follows the GEV distribution with parameters $\eta$, $\tau$ and $\xi$. The latter inequality is satisfied for any strictly positive $\delta$ such that $\delta < 1/(\xi \beta )-2$.□

#### A.8.2 Proof of proposition 2(iii)

##### Proof

Let *X* be the Brown–Resnick field associated with the semivariogram $\gamma _W(\varvec{x})=\Vert \varvec{x}\Vert ^2/2$, $\varvec{x} \in \mathbb {R}^2$, and with GEV parameters $\eta$, $\tau$ and $\xi \ne 0$, and $\beta \in \mathbb {N}_*$ such that $\beta \xi < 1/2$.

The field *X* is sample-continuous and thus measurable, which yields by Lemma 1 that $X^{\beta } \in \mathcal {C}$. Now, we have $X^{\beta }(\varvec{x})=D_{\beta , \eta , \tau , \xi }(Z(\varvec{x}))$, $\varvec{x} \in \mathbb {R}^2$, where *Z* is the simple Brown–Resnick field associated with the semivariogram just above, and $D_{\beta , \eta , \tau , \xi }$ is defined in (A15). In addition, by Lemma 2, $D_{\beta , \eta , \tau , \xi }$ satisfies the assumptions on the function *F* of Theorem 3 in Koch et al. (2019). Thus, the latter theorem yields that $X^{\beta }$ satisfies the CLT. This implies that

$$\begin{aligned} \int _{\mathbb {R}^2} \left| \textrm{Cov} \left( X^{\beta }(\varvec{0}), X^{\beta }(\varvec{x}) \right) \right| \textrm{d}\varvec{x} < \infty , \end{aligned}$$

which entails, using Theorem 3, that

$$\begin{aligned} \begin{aligned}&\int _{\mathbb {R}^2} \left( g_{\beta , \eta , \tau , \xi } \left( \Vert \varvec{x} \Vert \right) - \sum _{k_1=0}^{\beta } \sum _{k_2=0}^{\beta } B_{k_1, k_2, \beta , \eta , \tau , \xi } \ \Gamma (1-[\beta -k_1]\xi ) \Gamma (1-[\beta -k_2]\xi ) \right) \textrm{d}\varvec{x} \\&< \infty . \end{aligned} \end{aligned}$$

Since $g_{\beta , \eta , \tau , \xi }$ is strictly decreasing, this necessarily implies that

$$\begin{aligned} \lim _{h \rightarrow \infty } \left( g_{\beta , \eta , \tau , \xi } \left( h \right) - \sum _{k_1=0}^{\beta } \sum _{k_2=0}^{\beta } B_{k_1, k_2, \beta , \eta , \tau , \xi } \ \Gamma (1-[\beta -k_1]\xi ) \Gamma (1-[\beta -k_2]\xi ) \right) =0, \end{aligned}$$

i.e., (21).□

## Appendix B: Case of simple Brown–Resnick fields and $\beta <1/2$

This appendix explains that the results obtained in Sections 2.2 and 3.3 are similar if the Brown–Resnick field considered is simple and the power satisfies $\beta <1/2$. As standard Fréchet margins are rarely encountered in practice, the interest of this section mostly lies in a better understanding of some properties of simple Brown–Resnick fields and in possible applications to inference (using, e.g., the method of moments).

First we consider the dependence measure $\textrm{Corr} ( Z^{\beta }(\textbf{x}_1), Z^{\beta }(\textbf{x}_2))$, where $\{ Z(\textbf{x}) \}_{\textbf{x} \in \mathbb {R}^2}$ is a simple Brown–Resnick max-stable random field and $\beta < 1/2$. The condition $\beta \xi < 1/2$ with $\beta \in \mathbb {N}_*$ of (8) translates into $\beta < 1/2$; any negative value is allowed as simple max-stable fields are a.s. strictly positive. We introduce, for $\beta <1/2$,

$$\begin{aligned} I_{\beta }(h) = \left\{ \begin{array}{ll} \Gamma (1-2 \beta ) &{} \text{ if } \quad h=0, \\ \displaystyle \int _{0}^{\infty } \theta ^{\beta } \Big [ C_2(\theta ,h) \ C_1(\theta ,h)^{2 \beta -2} \ \Gamma (2-2 \beta ) \\ \qquad + C_3(\theta ,h) \ C_1(\theta ,h)^{2 \beta -1} \ \Gamma (1-2 \beta ) \Big ] \ \textrm{d}\theta &{} \text{ if } \quad h>0, \end{array} \right. \end{aligned}$$

which arises when setting $\beta _1=\beta _2$ in the function $I_{\beta _1, \beta _2}$ specified in (11). Denoting by $\gamma _W$ the semivariogram of *Z*, it follows from Theorem 1 and (4) that, for all $\textbf{x}_1, \textbf{x}_2 \in \mathbb {R}^2$ and $\beta < 1/2$, $\textrm{Cov} ( Z^{\beta }(\textbf{x}_1), Z^{\beta }(\textbf{x}_2) )=I_{\beta }(\sqrt{2 \gamma _W(\textbf{x}_2-\textbf{x}_1)}) - \left[ \Gamma (1- \beta ) \right] ^2$. Then $\textrm{Corr} ( Z^{\beta }(\textbf{x}_1), Z^{\beta }(\textbf{x}_2))$ (provided that $\beta \ne 0$) is readily derived and its behaviour is similar to the one we observed in Section 3.3 (not shown); for more details, see Figs. 3 and 4 in the unpublished work by Koch (2018).

We now investigate the function $I_{\beta }$ in further details. Very similar proofs as for Proposition 2 yield, for $\beta , \beta _1, \beta _2 <1/2$, that the functions $I_{\beta _1, \beta _2}$ defined in (11) and $I_{\beta }$ are strictly decreasing, $\lim _{h \rightarrow 0} I_{\beta }(h)=\Gamma (1-2 \beta )$ (implying that $I_{\beta }$ is continuous everywhere on $[0, \infty )$) and $\lim _{h \rightarrow \infty } I_{\beta }(h) = [\Gamma (1-\beta )]^2$. This entails that, for any $h \ge 0$, $\lim _{\beta \rightarrow -\infty } I_{\beta }(h)= \infty$. Figure 8, obtained using adaptive quadrature with a relative accuracy of $10^{-5}$, shows that the decrease of $I_{\beta }(h)$ for a given $\beta$ with respect to *h* is more and more pronounced when $\vert \beta \vert$ increases, and that, for *h* fixed, the absolute value of the slope of $I_{\beta }(h)$ increases very fast with $\vert \beta \vert$, in link with rapid divergence to $\infty$. Obviously, the behaviour of $\textrm{Cov} ( Z^{\beta }(\textbf{x}_1), Z^{\beta }(\textbf{x}_2))$ is similar; the same holds true for $I_{\beta _1, \beta _2}$.

## Appendix C: Performance of the Monte Carlo and empirical estimators

### C.1 Monte Carlo estimator

We numerically assess the performance of the Monte Carlo (MC) estimator of $\mathcal {D}_{X, \beta }(\varvec{x}_1, \varvec{x}_2)$ in various configurations, for *X* being a Brown–Resnick field with semivariogram (5). In each of these, we compute the relative errors of 100 estimates and display the resulting root mean square error (RMSE) in Table 3. We recall that the relative errors can be computed as the true value is known and given by (18). We set $\varvec{x}_1=(0, 0)'$ and $\psi =0.8$, and let $\beta$, $\xi$, $\eta$, $\tau$, $\kappa$, $\varvec{x}_2$, and *S* vary, where *S* denotes the number of simulations used in the MC estimation. For each combination of sites $(\varvec{x}_1, \varvec{x}_2)$, we need to simulate the Brown–Resnick field at only two sites and we therefore simulate *S* realizations from a bivariate Hüsler–Reiss vector with appropriate parameters; *S* is thus the number of independent pairs of observations. We do so using the rmev function of the mev R package (Belzile et al. 2022) which provides exact simulations.

Table 3 shows that the RMSE increases with the power $\beta$, and that this effect is stronger for strictly positive values of the shape parameter $\xi$. For $\xi >0$, the RMSE also increases with $\xi$. For $\xi >0$, the RMSE increases when the product $\beta \xi$ increases and becomes very large (up to $66.5 \%$ in the table) when it approaches 1/2; this is not surprising as $\mathcal {D}_{X, \beta }(\varvec{x}_1, \varvec{x}_2)$ is well defined if and only if $\beta \xi < 1/2$. For a given value of the product, the error is the largest when $\beta$ is the largest and $\xi$ the smallest. Moreover, the RMSE tends to increase when the distance between $\varvec{x}_1$ and $\varvec{x}_2$ increases and, consistently, to decrease when the range parameter $\kappa$ increases. It also increases when going from $\eta =25$ to $\eta =5$ or from $\tau =3$ to $\tau =10$, except for $\beta =1$ where there is no effect. When increasing *S* from $10^4$ to $10^5$, the RMSE decreases in almost all configurations. In the cases where $\beta \xi <1/4$, it is divided by a factor close to 3, which is expected according to the CLT. On the other hand, the decrease is very small for $\beta \xi > 1/4$, probably because the MC estimator has no variance and does not satisfy the CLT. Overall, the MC estimator performs rather well when the sites are not too far apart, $\kappa$ is large enough and $\beta \xi$ and $\beta$ are not too high. But, in some configurations, a large number of simulations would be needed to reach a reasonable accuracy.

**Table 3:** Root mean square error (computed using the relative differences) of the estimates in various configurations (in $\%$). Case (a) corresponds to $\eta =25$, $\tau =3$, $\kappa =3$; Case (b) to $\eta =5$, $\tau =3$, $\kappa =3$; Case (c) to $\eta =25$, $\tau =10$, $\kappa =3$; Case (d) to $\eta =25$, $\tau =3$, $\kappa =1$. The top panel corresponds to $S=10^4$ and the bottom one to $S=10^5$

		(a)			(b)			(c)			(d)		
		$\varvec{x}_2$			$\varvec{x}_2$			$\varvec{x}_2$			$\varvec{x}_2$		
$\xi$	$\beta$	(1, 1)’	(3, 3)’	(5, 5)’	(1, 1)’	(3, 3)’	(5, 5)’	(1, 1)’	(3, 3)’	(5, 5)’	(1, 1)’	(3, 3)’	(5, 5)’
-0.12	1	0.6	1.4	2.3	0.6	1.4	2.3	0.6	1.4	2.3	1.4	3.6	4.9
-0.12	2	0.6	1.4	2.4	0.7	1.7	2.9	0.7	1.6	2.7	1.4	3.8	5.1
-0.12	3	0.7	1.5	2.6	1.0	2.5	4.0	0.9	2.2	3.5	1.5	4.1	5.3
-0.12	4	0.7	1.7	2.8	1.5	3.6	5.5	1.2	3.0	4.6	1.7	4.5	5.8
-0.12	8	1.3	3.2	5.0	5.2	11.9	17.9	4.1	9.5	14.3	3.2	7.6	11.4
-0.24	1	0.7	1.4	2.3	0.7	1.4	2.3	0.7	1.4	2.3	1.4	3.5	5.0
-0.36	1	0.7	1.5	2.4	0.7	1.5	2.4	0.7	1.5	2.4	1.5	3.6	5.3
-0.48	1	0.8	1.7	2.6	0.8	1.7	2.6	0.8	1.7	2.6	1.7	3.7	5.7
-0.24	2	0.6	1.4	2.3	0.6	1.5	2.5	0.6	1.4	2.4	1.4	3.5	4.9
0.12	1	0.8	1.9	3.1	0.8	1.9	3.1	0.8	1.9	3.1	1.9	5.0	6.9
0.12	2	1.5	3.1	5.1	2.6	5.6	8.8	2.3	4.9	7.8	3.1	8.1	14.8
0.12	3	3.1	6.4	10.1	6.4	14.2	20.6	5.6	12.5	18.3	6.4	16.4	31.7
0.12	4	5.8	12.5	18.0	11.4	24.6	33.7	10.3	22.4	30.9	12.5	26.9	45.3
0.06	8	11.3	24.4	33.4	22.0	41.5	54.1	20.3	39.1	51.3	24.4	45.6	66.5
0.24	1	1.5	3.1	5.1	1.5	3.1	5.1	1.5	3.1	5.1	3.1	8.1	14.8
0.36	1	3.1	6.4	10.1	3.1	6.4	10.1	3.1	6.4	10.1	6.4	16.4	31.7
0.48	1	5.8	12.5	18.0	5.8	12.5	18.0	5.8	12.5	18.0	12.5	26.9	45.3
0.24	2	5.0	10.4	15.1	6.7	14.8	21.1	6.3	13.9	19.9	10.4	23.0	40.4
-0.12	1	0.2	0.4	0.6	0.2	0.4	0.6	0.2	0.4	0.6	0.4	1.1	1.9
-0.12	2	0.2	0.4	0.6	0.2	0.4	0.7	0.2	0.4	0.7	0.4	1.2	1.9
-0.12	3	0.2	0.4	0.7	0.3	0.6	1.0	0.3	0.5	0.9	0.4	1.3	1.9
-0.12	4	0.2	0.4	0.7	0.4	1.0	1.5	0.3	0.8	1.2	0.4	1.4	2.1
-0.12	8	0.4	0.9	1.3	1.6	4.7	6.9	1.2	3.5	5.0	0.9	2.3	3.6
-0.24	1	0.2	0.4	0.6	0.2	0.4	0.6	0.2	0.4	0.6	0.4	1.1	1.9
-0.36	1	0.2	0.5	0.7	0.2	0.5	0.7	0.2	0.5	0.7	0.5	1.2	2.1
-0.48	1	0.2	0.5	0.7	0.2	0.5	0.7	0.2	0.5	0.7	0.5	1.2	2.2
-0.24	2	0.2	0.4	0.6	0.2	0.4	0.6	0.2	0.4	0.6	0.4	1.1	1.9
0.12	1	0.2	0.5	0.8	0.2	0.5	0.8	0.2	0.5	0.8	0.5	1.4	2.3
0.12	2	0.4	1.2	1.6	0.8	2.7	3.7	0.7	2.3	3.1	1.2	2.7	4.2
0.12	3	1.2	4.3	5.8	3.6	10.6	16.4	3.1	9.3	14.3	4.3	10.3	14.1
0.12	4	4.0	11.0	16.9	8.2	22.1	31.3	7.5	20.3	29.2	11.0	25.6	34.8
0.06	8	9.1	23.9	33.4	16.2	40.2	50.5	15.1	37.9	48.3	23.9	47.6	64.9
0.24	1	0.4	1.2	1.6	0.4	1.2	1.6	0.4	1.2	1.6	1.2	2.7	4.2
0.36	1	1.2	4.3	5.8	1.2	4.3	5.8	1.2	4.3	5.8	4.3	10.3	14.1
0.48	1	4.0	11.0	16.9	4.0	11.0	16.9	4.0	11.0	16.9	11.0	25.6	34.8
0.24	2	3.3	9.0	13.6	4.7	12.9	19.7	4.4	12.2	18.7	9.0	21.1	28.1

### C.2 Empirical estimator

We numerically assess the performance of the empirical estimator of $\mathcal {D}_{X, \beta }(\varvec{x}_1, \varvec{x}_2)$ where $\varvec{x}_1 = (0, 0)'$ and $\varvec{x}_2=(1, 1)'$ or $(3, 3)'$, in similar configurations as in Section C.1. Instead of simulating *S* realizations from a bivariate Hüsler–Reiss distribution, we simulate 42 temporal observations of a Brown–Resnick field on a rectangle from $0 ^\circ$ to $6.25 ^\circ$ longitude and $0 ^\circ$ to $3 ^\circ$ latitude with a grid spacing of $0.25 ^\circ$ latitude and $0.25 ^\circ$ longitude; the number of observations and the grid are the same as in the case study (up to a translation for the grid). We take (5) as semivariogram with $\psi =0.8$, and let $\beta$, $\xi$, $\eta$, $\tau$, $\kappa$ vary. As before, we use the rmev function of the mev R package, which provides exact simulations of the Brown–Resnick field.

Since $\mathcal {D}_{X, \beta }(\varvec{x}_1, \varvec{x}_2)$ depends on $\varvec{x}_1$ and $\varvec{x}_2$ only through $\Vert \varvec{x}_2 - \varvec{x}_1 \Vert$, we compute the empirical estimate in three different ways:

- Method 1: we consider the observed time series at $\varvec{x}_1$ and $\varvec{x}_2$, take their power, and compute the empirical correlation. The time series are of length 42.
- Method 2: we create two time series by concatenating all time series at respectively grid points $\varvec{s}_i$ and $\varvec{s}_j$ such that $\varvec{s}_j - \varvec{s}_i= \varvec{x}_2$, and take the empirical correlation of their power. This allows benefiting from the fact that $\mathcal {D}_{X, \beta }(\varvec{x}_1, \varvec{x}_2)$ depends on $\varvec{x}_1$ and $\varvec{x}_2$ only through $\varvec{x}_2 - \varvec{x}_1$. For $\varvec{x}_2=(1, 1)'$ and $\varvec{x}_2=(3, 3)'$, respectively 198 and 14 pairs satisfy the condition, leading to time series of length 8316 and 588.
- Method 3: we build two time series by concatenating all times series at respectively grid points $\varvec{s}_i$ and $\varvec{s}_j$ such that $\Vert \varvec{s}_j - \varvec{s}_i \Vert = \Vert \varvec{x}_2 \Vert$, and take the empirical correlation of their power. This capitalizes on the fact that $\mathcal {D}_{X, \beta }(\varvec{x}_1, \varvec{x}_2)$ depends on $\varvec{x}_1$ and $\varvec{x}_2$ only through $\Vert \varvec{x}_2 - \varvec{x}_1 \Vert$. For $\varvec{x}_2=(1, 1)'$ and $\varvec{x}_2=(3, 3)'$, respectively 396 and 28 pairs satisfy the condition, leading to time series of length 16632 and 1176.

We repeat this procedure 100 times independently, derive the relative errors by comparison with the true value given by (18), and provide the resulting RMSE in Table 4. The comparison of the three methods yields insight about the benefits of concatenating dependent time series.

Table 4 shows, as expected, that the RMSE is generally smaller for the second method than for the first one. However, the decrease is rather low given the large increase of the number of observations (by a factor 198 and 14 for $\varvec{x}_2=(1, 1)'$ and $(3, 3)'$, respectively), and this comes from the strong spatial dependence which dramatically reduces the effective sample sizes of the formed time series. The relative decrease is larger for $\kappa =1$ than for $\kappa =3$ because the associated spatial dependence is weaker. Moreover, we observe an increase in some configurations where $\beta \xi > 1/4$, which is due to a slow convergence in link with the absence of variance and inapplicability of the CLT; see the related comments in the assessment of the MC method.

The RMSEs obtained with Methods 2 and 3 are comparable. Method 3 involves twice as many observations, but the difference in effective sample size is probably low owing to the strong spatial dependence mentioned above. Also, the number of independent replications used to compute the RMSE (100) is perhaps too low for a systematic decline to be visible. Nevertheless, we do observe a rather systematic decrease (although slight) for $\kappa =1$, because the associated spatial dependence is weaker.

In terms of evolution of the RMSE with respect to the various parameters, the conclusions are similar to those obtained for the MC estimator in Section C.1, but the differences between the RMSEs in the best (e.g., $\xi = -0.12$ and $\beta =1$) and worst (e.g., $\xi = 0.06$ and $\beta =8$) cases are much smaller than in the study of Section C.1. This is due to much lower effective sample sizes that prevent us from seeing the effects of fast and slow convergence in the respective configurations. Overall, the values of RMSE are large and so the use of the exact formula is recommended if the model is well-specified.

**Table 4:** RMSE (computed using the relative differences) of the estimates in various configurations (in $\%$). Cases (a), (b), (c), (d) are the same as in Table 3. The three panels correspond, from top to bottom, to Methods 1, 2, and 3

		(a)		(b)		(c)		(d)	
		$\varvec{x}_2$		$\varvec{x}_2$		$\varvec{x}_2$		$\varvec{x}_2$	
$\xi$	$\beta$	(1, 1)’	(3, 3)’	(1, 1)’	(3, 3)’	(1, 1)’	(3, 3)’	(1, 1)’	(3, 3)’
-0.12	1	10.3	21.8	10.3	21.8	10.3	21.8	19.2	58.2
-0.12	2	10.2	22.0	10.9	24.8	10.5	23.5	19.8	58.6
-0.12	3	10.3	22.7	13.8	29.8	12.3	27.3	21.0	59.8
-0.12	4	10.7	23.8	17.5	35.1	15.0	31.6	22.7	61.4
-0.12	8	14.5	30.7	31.1	51.8	27.0	47.1	31.8	70.0
-0.24	1	10.8	22.5	10.8	22.5	10.8	22.5	19.4	58.8
-0.36	1	11.6	24.0	11.6	24.0	11.6	24.0	20.5	60.9
-0.48	1	12.7	26.0	12.7	26.0	12.7	26.0	22.2	64.6
-0.24	2	10.3	21.9	10.1	23.1	10.0	22.3	19.1	58.2
0.12	1	10.9	23.8	10.9	23.8	10.9	23.8	22.3	60.7
0.12	2	12.0	26.2	15.1	31.6	14.0	29.9	25.5	62.8
0.12	3	13.7	29.2	21.2	39.8	18.9	36.9	29.5	65.0
0.12	4	16.0	32.7	27.0	46.7	24.0	43.3	34.1	67.1
0.06	8	22.1	41.0	39.9	60.5	36.4	57.0	44.9	77.0
0.24	1	12.0	26.2	12.0	26.2	12.0	26.2	25.5	62.8
0.36	1	13.7	29.2	13.7	29.2	13.7	29.2	29.5	65.0
0.48	1	16.0	32.7	16.0	32.7	16.0	32.7	34.1	67.1
0.24	2	14.7	30.4	18.6	36.4	17.4	34.7	31.1	64.3
-0.12	1	7.0	16.8	7.0	16.8	7.0	16.8	12.3	38.7
-0.12	2	7.5	17.5	9.0	20.5	8.4	19.3	13.0	39.6
-0.12	3	8.1	18.5	11.6	25.2	10.3	22.8	13.9	41.2
-0.12	4	8.7	19.7	14.6	30.2	12.6	26.9	15.0	43.2
-0.12	8	12.2	26.0	30.1	47.5	25.0	42.7	20.5	52.6
-0.24	1	6.4	16.3	6.4	16.3	6.4	16.3	11.5	38.3
-0.36	1	6.2	16.3	6.2	16.3	6.2	16.3	11.1	39.2
-0.48	1	6.1	16.7	6.1	16.7	6.1	16.7	11.0	40.8
-0.24	2	6.7	16.6	7.9	18.7	7.4	17.7	11.9	38.2
0.12	1	8.8	19.6	8.8	19.6	8.8	19.6	15.3	43.5
0.12	2	10.1	22.0	13.1	27.3	12.1	25.6	17.4	46.7
0.12	3	12.0	25.1	20.2	36.6	17.7	33.5	20.7	49.8
0.12	4	15.1	29.5	29.1	45.3	25.3	41.4	26.4	53.6
0.06	8	23.6	39.2	53.4	62.5	47.7	58.6	40.7	63.4
0.24	1	10.1	22.0	10.1	22.0	10.1	22.0	17.4	46.7
0.36	1	12.0	25.1	12.0	25.1	12.0	25.1	20.7	49.8
0.48	1	15.1	29.5	15.1	29.5	15.1	29.5	26.4	53.6
0.24	2	13.5	27.2	17.8	33.4	16.5	31.6	23.7	50.8
-0.12	1	6.9	16.9	6.9	16.9	6.9	16.9	12.0	34.6
-0.12	2	7.4	17.8	9.0	20.7	8.4	19.6	12.7	36.0
-0.12	3	8.0	18.8	11.7	25.3	10.4	23.0	13.7	37.8
-0.12	4	8.7	20.0	14.8	30.2	12.7	26.9	14.7	40.0
-0.12	8	12.3	26.3	30.9	49.9	25.6	44.2	20.3	49.4
-0.24	1	6.3	16.1	6.3	16.1	6.3	16.1	11.2	33.6
-0.36	1	5.9	15.7	5.9	15.7	5.9	15.7	10.8	33.6
-0.48	1	5.9	15.8	5.9	15.8	5.9	15.8	10.7	34.4
-0.24	2	6.6	16.5	7.8	18.8	7.3	17.8	11.5	33.9
0.12	1	8.8	20.1	8.8	20.1	8.8	20.1	15.0	40.2
0.12	2	10.1	22.4	13.2	27.7	12.1	26.0	17.2	43.4
0.12	3	12.0	25.7	20.5	38.2	17.9	34.7	20.6	46.8
0.12	4	15.2	30.6	29.7	48.6	25.7	44.2	26.5	51.3
0.06	8	24.0	42.0	54.7	69.7	48.8	65.1	41.1	62.1
0.24	1	10.1	22.4	10.1	22.4	10.1	22.4	17.2	43.4
0.36	1	12.0	25.7	12.0	25.7	12.0	25.7	20.6	46.8
0.48	1	15.2	30.6	15.2	30.6	15.2	30.6	26.5	51.3
0.24	2	13.6	28.0	18.0	34.9	16.7	32.9	23.6	48.4

## Footnotes

1 Throughout the paper, stationarity refers to strict stationarity.

2 Throughout, when applied to random fields, the adjective “measurable” means “jointly measurable”.

3 As shown in the aforementioned ongoing project about spatial risk measures, the assumption of strict positiveness everywhere is reasonable.

## References

[CR1] Asadi, P., Davison, A.C., Engelke, S.: Extremes on river networks. Ann. Appl. Stat. **9**(4), 2023–2050 (2015). 10.1214/15-AOAS863 10.1214/15-AOAS863

[CR2] Belzile, L., Wadsworth, J.L., Northrop, P.J., Grimshaw, S.D., Zhang, J., Stephens, M.A., Huser, R.: mev: Multivariate Extreme Value Distributions. R package version **1**, 14 (2022)

[CR3] Berg, C., Christensen, J.P.R., Ressel, P.: Harmonic Analysis on Semigroups: Theory of Positive Definite and Related Functions. Springer New-York (1984). 10.1007/978-1-4612-1128-0 10.1007/978-1-4612-1128-0

[CR4] Billingsley, P.: Convergence of Probability Measures. John Wiley & Sons (1999). 10.1002/9780470316962 10.1002/9780470316962

[CR5] Blanchet, J., Lehning, M.: Mapping snow depth return levels: smooth spatial modeling versus station interpolation. Hydrol. Earth Syst. Sci. **14**(12), 2527–2544 (2010). 10.5194/hess-14-2527-2010 10.5194/hess-14-2527-2010

[CR6] Brown, B.M., Resnick, S.I.: Extreme values of independent stochastic processes. J. Appl. Probab. **14**(4), 732–739 (1977). 10.2307/3213346 10.2307/3213346

[CR7] Burton, T.L., Jenkins, N., Bossanyi, E., Sharpe, D., Michael, G.: Wind Energy Handbook. John Wiley & Sons (2021). 10.1002/9781119451143 10.1002/9781119451143

[CR8] Ceppi, P., Della-Marta, P.M., Appenzeller, C.: Extreme value analysis of wind speed observations over Switzerland. Arbeitsberichte der MeteoSchweiz **219**, (2008). https://www.meteoswiss.admin.ch/home/services-and-publications/publications.subpage.html/en/data/publications/2008/1/extreme-value-analysis-of-wind-speed-observations-over-switzerla.html

[CR9] Cont, R.: Empirical properties of asset returns: stylized facts and statistical issues. Quant. Finan. **1**(2), 223–236 (2001). 10.1080/713665670 10.1080/713665670

[CR10] Cooley, D., Naveau, P., Poncet, P.: Variograms for spatial max-stable random fields. Dependence in Probability and Statistics, Lecture Notes in Statistics. vol. 187, pp. 373–390 (2006). 10.1007/0-387-36062-X_17

[CR11] Davison, A.C., Padoan, S.A., Ribatet, M.: Statistical modeling of spatial extremes. Stat. Sci. **27**(2), 161–186 (2012). 10.1214/11-STS376 10.1214/11-STS376

[CR12] de Fondeville, R., Davison, A.C.: High-dimensional peaks-over-threshold inference. Biometrika. **105**(3), 575–592 (2018). 10.1093/biomet/asy026 10.1093/biomet/asy026

[CR13] de Haan, L.: A spectral representation for max-stable processes. Ann. Probab. **12**(4), 1194–1204 (1984). 10.1214/aop/1176993148 10.1214/aop/1176993148

[CR14] de Haan, L., Ferreira, A.: Extreme Value Theory: An Introduction. Springer-Verlag, New York. (2006). 10.1007/0-387-34471-3 10.1007/0-387-34471-3

[CR15] de Haan, L., Resnick, S.I.: Limit theory for multivariate sample extremes. Z. Wahrscheinlichkeitstheorie Verw. Gebiete. **40**, 317–337 (1977). 10.1007/BF00533086 10.1007/BF00533086

[CR16] Della-Marta, P.M., Mathis, H., Frei, C., Liniger, M.A., Appenzeller, C.: Extreme wind storms over Europe: statistical analyses of ERA-40. Arbeitsberichte der MeteoSchweiz **216**, (2007). https://www.meteoswiss.admin.ch/home/services-and-publications/publications.subpage.html/en/data/publications/2007/1/extreme-wind-storms-over-europe--statistical-analyses-of-era-40.html

[CR17] Denuit, M., Dhaene, J., Goovaerts, M., Kaas, R.: Actuarial Theory for Dependent Risks: Measures, Orders and Models. John Wiley & Sons (2005). 10.1002/0470016450

[CR18] Dhaene, J., Goovaerts, M.J.: Dependency of risks and stop-loss order. ASTIN Bull. **26**(02), 201–212 (1996). 10.2143/AST.26.2.563219 10.2143/AST.26.2.563219

[CR19] Ding, Z., Granger, C.W.J., Engle, R.F.: A long memory property of stock market returns and a new model. J. Empir. Finan. **1**(1), 83–106 (1993). 10.1016/0927-5398(93)90006-D 10.1016/0927-5398(93)90006-D

[CR20] Donat, M.G., Pardowitz, T., Leckebusch, G.C., Ulbrich, U., Burghoff, O.: High-resolution refinement of a storm loss model and estimation of return periods of loss-intensive storms over Germany. Nat. Hazards Earth Syst. Sci. **11**(10), 2821–2833 (2011). 10.5194/nhess-11-2821-2011 10.5194/nhess-11-2821-2011

[CR21] Einmahl, J.H.J., Kiriliouk, A., Krajina, A., Segers, J.: An M-estimator of spatial tail dependence. J. R. Stat. Soc. Ser. B Stat. Methodol. **78**(1), 275–298 (2016). 10.1111/rssb.12114 10.1111/rssb.12114

[CR22] Emanuel, K.: Increasing destructiveness of tropical cyclones over the past 30 years. Nature **436**(4), 686–688 (2005). 10.1038/nature03906 16056221 10.1038/nature03906

[CR23] Hinkel, J., Lincke, D., Vafeidis, A.T., Perrette, M., Nicholls, R.J., Tol, R.S.J., Levermann, A.: Coastal flood damage and adaptation costs under 21st century sea-level rise. Proc. Natl. Acad. Sci. USA **111**(9), 3292–3297 (2014). 10.1073/pnas.1222469111 24596428 10.1073/pnas.1222469111 PMC3948227

[CR24] Huang, Z., Rosowsky, D.V., Sparks, P.R.: Long-term hurricane risk assessment and expected damage to residential structures. Reliab. Eng. Syst. Saf. **74**(3), 239–249 (2001). 10.1016/S0951-8320(01)00086-2 10.1016/S0951-8320(01)00086-2

[CR25] Huser, R., Davison, A.C.: Composite likelihood estimation for the Brown–Resnick process. Biometrika **100**(2), 511–518 (2013). 10.1093/biomet/ass089 10.1093/biomet/ass089

[CR26] Hüsler, J., Reiss, R.D.: Maxima of normal random vectors: between independence and complete dependence. Statist. Probab. Lett. **7**(4), 283–286 (1989). 10.1016/0167-7152(89)90106-5 10.1016/0167-7152(89)90106-5

[CR27] Kabluchko, Z., Schlather, M., de Haan, L.: Stationary max-stable fields associated to negative definite functions. Ann. Probab. **37**(5), 2042–2065 (2009). 10.1214/09-AOP455 10.1214/09-AOP455

[CR28] Kantha, L.: Tropical cyclone destructive potential by integrated kinetic energy. Bull. Am. Meteorol. Soc. **89**(2), 219–221 (2008). https://www.jstor.org/stable/26216782

[CR29] Klawa, M., Ulbrich, U.: A model for the estimation of storm losses and the identification of severe winter storms in Germany. Nat. Hazards Earth Syst. Sci. **3**(6), 725–732 (2003). 10.5194/nhess-3-725-2003 10.5194/nhess-3-725-2003

[CR30] Koch, E.: Tools and Models for the Study of some Spatial and Network Risks: Application to Climate Extremes and Contagion in Finance PhD thesis. ISFA, Université Claude Bernard Lyon **1**, (2014). https://tel.archives-ouvertes.fr/tel-01284995/document

[CR31] Koch, E.: Spatial risk measures and applications to max-stable processes. Extremes **20**(3), 635–670 (2017). 10.1007/s10687-016-0274-0 10.1007/s10687-016-0274-0

[CR32] Koch, E.: Spatial risk measures induced by powers of max-stable random fields (2018). arXiv preprint arXiv:1804.05694v1. https://arxiv.org/abs/1804.05694v1

[CR33] Koch, E. (2019a). Extremal dependence and spatial risk measures for insured losses due to extreme winds. arXiv preprint arXiv:1804.05694v2. https://arxiv.org/abs/1804.05694v2

[CR34] Koch, E.: Spatial risk measures and rate of spatial diversification. Risks **7**(2), 52 (2019b). 10.3390/risks7020052 10.3390/risks7020052

[CR35] Koch, E., Dombry, C., Robert, C.Y.: A central limit theorem for functions of stationary mixing max-stable random fields on . Stoch. Proc. Appl. **129**(9), 3406–3430 (2019). 10.1016/j.spa.2018.09.014 10.1016/j.spa.2018.09.014

[CR36] Koch, E., Robert, C.Y.: Stochastic derivative estimation for max-stable random fields. Eur. J. Oper. Res. **302**(2), 575–588 (2022). 10.1016/j.ejor.2021.12.026 10.1016/j.ejor.2021.12.026

[CR37] Lamb, H., Frydendahl, K.: Historic Storms of the North Sea. Cambridge University Press, British Isles and Northwest Europe (1991)

[CR38] Naveau, P., Guillou, A., Cooley, D., Diebolt, J.: Modelling pairwise dependence of maxima in space. Biometrika **96**(1), 1–17 (2009). 10.1093/biomet/asp001 10.1093/biomet/asp001

[CR39] Opitz, T.: Extremal *t* processes: elliptical domain of attraction and a spectral representation. J. Multivar. Anal. **122**, 409–413 (2013). 10.1016/j.jmva.2013.08.008 10.1016/j.jmva.2013.08.008

[CR40] Padoan, S.A., Ribatet, M., Sisson, S.A.: Likelihood-based inference for max-stable processes. J. Am. Stat. Assoc. **105**(489), 263–277 (2010). 10.1198/jasa.2009.tm08577 10.1198/jasa.2009.tm08577

[CR41] Pinto, J.G., Fröhlich, E.L., Leckebusch, G.C., Ulbrich, U.: Changing European storm loss potentials under modified climate conditions according to ensemble simulations of the ECHAM5/MPI-OM1 GCM. Nat. Hazards Earth Syst. Sci. **7**(1), 165–175 (2007). 10.5194/nhess-7-165-2007 10.5194/nhess-7-165-2007

[CR42] Powell, M.D., Reinhold, T.A.: Tropical cyclone destructive potential by integrated kinetic energy. Bull. Am. Meteorol. Soc. **88**(4), 513–526 (2007). 10.1175/BAMS-88-4-513 10.1175/BAMS-88-4-513

[CR43] Prahl, B.F., Rybski, D., Boettle, M., Kropp, J.P.: Damage functions for climate-related hazards: unification and uncertainty analysis. Nat. Hazards Earth Syst. Sci. **16**(5), 1189–1203 (2016). 10.5194/nhess-16-1189-2016 10.5194/nhess-16-1189-2016

[CR44] Prahl, B.F., Rybski, D., Burghoff, O., Kropp, J.P.: Comparison of storm damage functions and their performance. Nat. Hazards Earth Syst. Sci. **15**, 769–788 (2015). 10.5194/nhess-15-769-2015 10.5194/nhess-15-769-2015

[CR45] Prahl, B.F., Rybski, D., Kropp, J.P., Burghoff, O., Held, H.: Applying stochastic small-scale damage functions to German winter storms. Geophys. Res. Lett. **39**(6), (2012). 10.1029/2012GL050961

[CR46] Prettenthaler, F., Albrecher, H., Köberl, J., Kortschak, D.: Risk and insurability of storm damages to residential buildings in Austria. Geneva Pap. Risk Insur. Issues Pract. **37**(2), 340–364 (2012). 10.1057/gpp.2012.15 10.1057/gpp.2012.15

[CR47] Ribatet, M.: Spatial extremes: Max-stable processes at work. J. Soc. Franç. Stat. **154**(2), 156–177 (2013). http://www.numdam.org/item/JSFS_2013_154_2_156_0/

[CR48] Ribatet, M.: SpatialExtremes: Modelling Spatial Extremes. R package version 2.0-9 (2020). https://CRAN.R-project.org/package=SpatialExtremes

[CR49] Schlather, M., Tawn, J.A.: A dependence measure for multivariate and spatial extreme values: properties and inference. Biometrika **90**(1), 139–156 (2003). 10.1093/biomet/90.1.139 10.1093/biomet/90.1.139

[CR50] Simiu, E., Scanlan, R.H.: Wind Effects on Structures: Fundamentals and Applications to Design. John Wiley & Sons (1996)

[CR51] Smith, R.L.: Max-stable processes and spatial extremes. University of Surrey (1990). https://www.rls.sites.oasis.unc.edu/postscript/rs/spatex.pdf

[CR52] Varin, C., Vidoni, P.: A note on composite likelihood inference and model selection. Biometrika **92**(3), 519–528 (2005). 10.1093/biomet/92.3.519 10.1093/biomet/92.3.519

